# Tethering of the Conserved piggyBac Transposase Fusion Protein CSB-PGBD3 to Chromosomal AP-1 Proteins Regulates Expression of Nearby Genes in Humans

**DOI:** 10.1371/journal.pgen.1002972

**Published:** 2012-09-27

**Authors:** Lucas T. Gray, Kimberly K. Fong, Thomas Pavelitz, Alan M. Weiner

**Affiliations:** Department of Biochemistry, School of Medicine, University of Washington, Seattle, Washington, United States of America; Stanford University School of Medicine, United States of America

## Abstract

The CSB-PGBD3 fusion protein arose more than 43 million years ago when a 2.5-kb piggyBac 3 (PGBD3) transposon inserted into intron 5 of the Cockayne syndrome Group B (CSB) gene in the common ancestor of all higher primates. As a result, full-length CSB is now coexpressed with an abundant CSB-PGBD3 fusion protein by alternative splicing of CSB exons 1–5 to the PGBD3 transposase. An internal deletion of the piggyBac transposase ORF also gave rise to 889 dispersed, 140-bp MER85 elements that were mobilized in trans by PGBD3 transposase. The CSB-PGBD3 fusion protein binds MER85s *in vitro* and induces a strong interferon-like innate antiviral immune response when expressed in CSB-null UVSS1KO cells. To explore the connection between DNA binding and gene expression changes induced by CSB-PGBD3, we investigated the genome-wide DNA binding profile of the fusion protein. CSB-PGBD3 binds to 363 MER85 elements *in vivo*, but these sites do not correlate with gene expression changes induced by the fusion protein. Instead, CSB-PGBD3 is enriched at AP-1, TEAD1, and CTCF motifs, presumably through protein–protein interactions with the cognate transcription factors; moreover, recruitment of CSB-PGBD3 to AP-1 and TEAD1 motifs correlates with nearby genes regulated by CSB-PGBD3 expression in UVSS1KO cells and downregulated by CSB rescue of mutant CS1AN cells. Consistent with these data, the N-terminal CSB domain of the CSB-PGBD3 fusion protein interacts with the AP-1 transcription factor c-Jun and with RNA polymerase II, and a chimeric CSB-LacI construct containing only the N-terminus of CSB upregulates many of the genes induced by CSB-PGBD3. We conclude that the CSB-PGBD3 fusion protein substantially reshapes the transcriptome in CS patient CS1AN and that continued expression of the CSB-PGBD3 fusion protein in the absence of functional CSB may affect the clinical presentation of CS patients by directly altering the transcriptional program.

## Author Summary

Cockayne syndrome (CS) is a terrible and ultimately fatal childhood progeria often caused by mutations in the CSB chromatin remodeling and DNA repair protein. A piggyBac transposable element invaded the CSB gene about 43 million years ago, before humans diverged from marmosets. However, this last common ancestor “domesticated” the “selfish” invader, and our CSB locus now encodes both the original CSB protein and a novel CSB-piggyBac fusion protein joining the first third of the CSB protein to the piggyBac transposase. Although likely to be advantageous in health, expression of the conserved fusion protein in cells lacking any CSB-related protein induces a strong interferon response that may explain the brain calcifications seen in advanced CS. To determine whether continued expression of the fusion protein in CS patients affects the severity or heterogeneity of the disease, we identified all genomic binding sites for the fusion protein experimentally. We find that the fusion protein is tethered by protein–protein interactions to at least three transcription factor binding motifs on DNA (AP-1, TEAD1, and CTCF motifs). The tethered fusion protein regulates nearby genes, including some that may induce the interferon response. Our data suggest that drugs or biologicals targeting innate immunity and inflammation may benefit CS patients.

## Introduction

Cockayne syndrome (CS) is a neurodevelopmental disorder most often caused by loss of functional CSB or CSA protein (OMIM #133540 or #216400) [1]. CSB is a SWI/SNF2-like ATPase and chromatin remodeling protein that plays a key role in transcription-coupled nucleotide excision repair (TC-NER) of helix-distorting DNA lesions. When RNA polymerase II (RNAPII) stalls at a site of DNA damage, CSB is among the first proteins to bind [2]–[4] and is required to recruit other NER factors including CSA and the TFIIH complex containing the XPB and XPD helicases [5]–[7]. CSB is also known to activate RNA polymerase I (RNAPI) transcription of ribosomal RNA [8], and to induce changes in gene expression resembling those caused by chromatin remodeling and histone modification [9].

**Figure 1:**
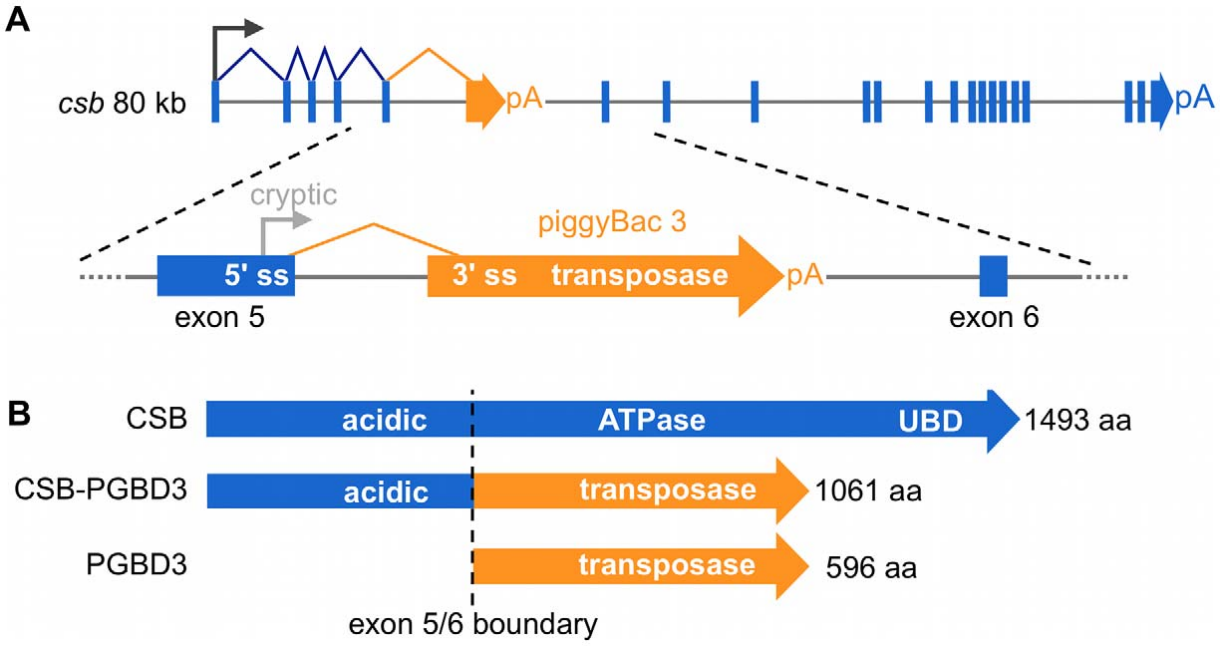
The CSB-PGBD3 fusion protein is abundantly expressed by alternative splicing and polyadenylation of the CSB transcript. (A) The CSB-PGBD3 fusion protein is expressed by alternative splicing of CSB exons 1–5 to the PGBD3 transposase 3′ splice acceptor site, whereas solitary PGBD3 transposase is expressed from a cryptic promoter in CSB exon 5. (B) As a result, the primate CSB locus generates three proteins: full-length CSB, the CSB-PGBD3 fusion protein, and solitary PGBD3 transposase. pA, polyadenylation signal; 5′ ss, 5′ splice donor site; 3′ss, 3′ splice acceptor site.

We recently discovered a domesticated PGBD3 transposon (piggyBac transposable element-derived 3) that inserted into intron 5 of the CSB gene at least 43 Mya in the common ancestor of marmoset and humans. As a result, primate CSB genes including our own now generate both full length CSB (coding exons 2–21) and — by alternative splicing and polyadenylation — a CSB-PGBD3 fusion protein that joins the N-terminal domain of CSB (coding exons 2–5) to the intact PGBD3 transposase [10]. CSB-PGBD3 is startlingly well conserved from marmoset to humans, whereas four other identifiable copies of the PGBD3 transposon elsewhere in the human genome have all decayed into pseudogenes (PGBD3P1-4). The PGBD3 transposon contains a 5′ splice acceptor site just upstream of the transposase ORF and a polyadenylation signal downstream of the ORF that allow alternative splicing of CSB exon 5 to the intact transposase without precluding continued expression of full length CSB (Figure 1). In fact, the insertion of PGBD3 expanded the repertoire of the CSB locus from one protein to three: full length CSB, the more abundant CSB-PGBD3 fusion protein, and most abundant of all, the intact PGBD3 transposase transcribed from a cryptic promoter near the 3′ end of CSB exon 5 [10]. Coexpression of the CSB-PGBD3 fusion protein with CSB initially suggested that the fusion protein might contribute to or modulate CS disease [10]; however, mutations that cause CS are distributed across the entire length of the CSB gene (except in the PGBD3 transposon) and no consistent clinical differences have been observed between CS patients with CSB mutations in coding exons 2–5 (many of whom do not make the CSB-PGBD3 fusion protein) and patients with mutations in exons 6–21 (who continue to make the CSB-PGBD3 fusion protein) [1].

Unlike the ATPase domain (CSB exons 6–21), the function of the N-terminal domain (coding exons 2–5) shared by CSB and the CSB-PGBD3 fusion protein is not yet well understood (Figure 1). The only recognizable motif in exons 2–5 is a highly acidic domain between E356 and E403 containing 25 aspartates and glutamates, but this domain does not appear to be essential for recovery of RNA synthesis following UV damage [11], [12]. Interestingly, the N-terminus autoinhibits association of CSB with chromatin in both normal and UV-irradiated cells, and ATP hydrolysis is required for relief of inhibition [13]. The isolated N-terminal domain has also been shown to interfere with transcription and repair: Truncated CSB protein expressed in the patient-derived cell line CS1AN represses elongation by RNAPI [14] and the N-terminus of CSB interacts with topoisomerase I (Top1) to inhibit repair of Top1 adducts both as part of the CSB-PGBD3 fusion protein and independently [15].

We have recently shown that expression of the CSB-PGBD3 fusion protein in CSB-null UVSS1KO cells induces a strong transcriptional response dominated by an interferon-like innate antiviral immune response that may be driven by upregulation of the STAT1, STAT2, and IRF9 components of the heterotrimeric transcription factor ISGF3 (interferon-stimulated gene factor 3) [16]. As might be expected from conservation of the CSB-PGBD3 fusion protein for over 43 My, the interferon-like response induced by CSB-PGBD3 is dramatically repressed by coexpression of full-length CSB, and is not induced by CSB alone. However, the mechanism by which the CSB-PGBD3 fusion protein induces the interferon-like response, and CSB represses it, are still unclear.

The CSB-PGBD3 fusion protein may affect RNAPII gene expression through both global and local mechanisms. Globally, CSB-PGBD3 may modulate CSB functions by interacting with complexes that normally contain functional CSB; this could explain how the fusion protein modulates DNA repair without inducing or repressing transcription of known DNA repair factors [16]. CSB-PGBD3 may also affect RNAPII transcription locally by binding to dispersed DNA elements called MER85s, thereby regulating expression of nearby genes.

PGBD3, like many autonomous mobile elements, has given rise to a family of internally-deleted, nonautonomous elements that can be mobilized by the PGBD3 transposase. These 140 bp MER85 elements retain about 100 bp from the 5′ end of PGBD3, and about 40 bp from the 3′ end, but have lost the transposase ORF along with the upstream 5′ SS and the downstream poly(A) site. We have identified 889 MER85 elements dispersed throughout the human genome, most of which include 13 bp terminal inverted repeats (TIRs) that are required by the PGBD3 transposase for excision and reinsertion into TTAA target sites. We have also demonstrated that MER85 elements bind PGBD3 and CSB-PGBD3 *in vitro* [16]. Thus, CSB-PGBD3 may enable MER85s to recruit the N-terminus of CSB to specific genomic loci where it can affect local chromatin structure or recruit transcription and repair factors.

We wish to understand why the CSB-PGBD3 fusion protein is so well conserved, and to determine what roles it may play in health and CS disease. Here, we explore the connection between the genome-wide DNA binding profile of CSB-PGBD3 and transcriptional regulation in UVSS1KO cells. As expected, we find that CSB-PGBD3 binds directly *in vivo* to many MER85 elements throughout the genome. Surprisingly, we also find that CSB-PGBD3 binds indirectly to TRE motifs (tumor promoting antigen response elements) recognized by AP-1 family (activating protein-1) transcription factors, as well as to motifs for the TEAD1 (TEA domain family member 1) and CTCF (CCCTC-binding factor) transcription factors. We show that CSB-PGBD3 physically interacts with the AP-1 protein c-Jun, and that genes upregulated by CSB-PGBD3 correlate with binding of CSB-PGBD3 to nearby TRE motifs but not with binding to MER85 elements. We also show that CSB-PGBD3 interacts with RNAPII (RNA polymerase II), and that interactions with RNAPII and c-Jun are both mediated primarily by the N-terminal CSB domain of CSB-PGBD3. Thus despite the ability of the CSB-PGBD3 fusion protein to bind specifically to MER85s both *in vitro* and *in vivo*, binding does not appear to have widespread transcriptional consequences. In contrast, binding of the CSB-PGBD3 fusion protein to TRE motifs through protein-protein interactions with c-Jun and possibly other AP-1 family members correlates with genes involved in angiogenesis [17], [18], innate immunity [19], and the Smad2/3 and TGF-beta pathways [20], demonstrating that the CSB-PGBD3 protein modulates a preexisting AP-1-based regulatory network. Whether these regulatory effects were responsible for initial fixation of the CSB-PGBD3 fusion protein in the common ancestor of humans and marmoset 43 Mya, or whether these regulatory effects have evolved over time, remains to be seen.

## Results

### The CSB-PGBD3 fusion protein binds to the 5′ end of MER85 elements *in vivo*

**Figure 2:**
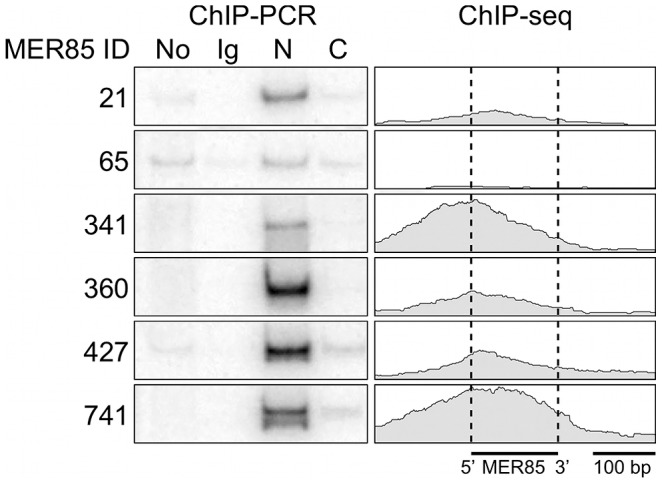
The CSB-PGBD3 fusion protein binds to MER85 elements *in vivo*. (*left*) ChIP-PCR in wild type HT1080 cells using antibodies for the N-terminus of CSB pulls down 6 representative MER85 elements with good matches to the Repbase consensus. N-terminal antibodies pull down both CSB-PGBD3 fusion protein and full-length CSB, whereas C-terminal antibodies pull down full-length CSB only (LTG unpublished). No, no antibody control; Ig, anti-mouse IgG nonspecific antibody control; N, CSB N-terminal antibody; C, CSB C-terminal antibody (*right*) Paired-end ChIP-seq shows enrichment for the same five out of six MER85 elements in CSB-null UVSS1KO cells stably expressing the CSB-PGBD3 fusion protein. Table S1 gives the positions and sequences of all MER85 elements. The 5′ and 3′ ends of MER85s are defined as the same orientation as the transposase ORF in parental PGBD3 elements.

Using a panel of six highly conserved MER85s with >90% identity to the Repbase MER85 consensus [21], we found previously that both the CSB-PGBD3 fusion protein and solitary PGBD3 transposase can bind MER85 elements *in vitro* [16]. To extend these results to living cells, we performed ChIP-PCR (chromatin immunoprecipitation followed by radiolabeled PCR) using human euploid HT1080 fibrosarcoma cells, genomic primers for the same six MER85 elements, and antibodies directed against the N- or C-terminus of CSB (Figure 2). We first confirmed that in HT1080 cells, which are wild-type for CSB and CSB-PGBD3, antibody against the N-terminus of CSB immunoprecipitated both CSB and CSB-PGBD3, whereas antibody against the C-terminus brought down CSB alone (data not shown). ChIPs with antibody against the N-terminus of CSB enriched for 5 of 6 MER85 elements *in vivo* including all 4 elements that shifted in the electrophoretic mobility shift assay (EMSA) [16]; ChIPs using antibody against the C-terminus or nonspecific antibody did not enrich for any of the six MER85s (Figure 2).

**Figure 3:**
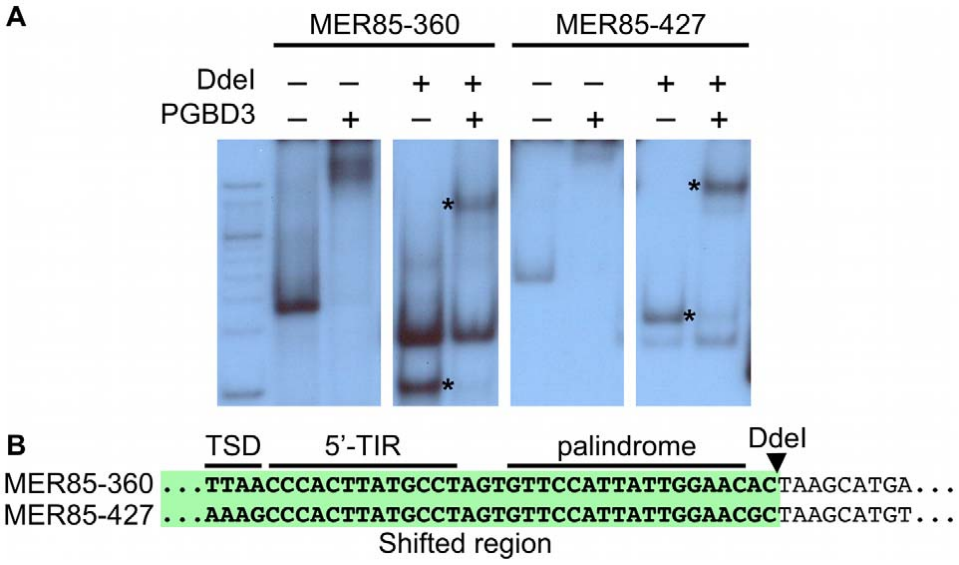
The PGBD3 transposase binds to the 5′ end of MER85s *in vitro*. (A) An electrophoretic mobility shift assay (EMSA) using MER85s and MER85 DdeI restriction fragments. MER85-360 and MER85-427 were excised from plasmid clones using BamHI and EcoRI, then digested with DdeI or left intact. The restriction fragments were end-labeled and mixed with purified PGBD3 transposase. Restriction fragments derived from the 5′ end of each MER85 are marked by an asterisk. (B) Partial sequences of MER85-360 and MER85-427 with the DdeI restriction site indicated. The 5′ MER85 sequences that shifted upon incubation with transposase are highlighted in green. TSD, target site duplication; 5′-TIR, 5′ terminal inverted repeat.

To explore the DNA sequence requirements for CSB-PGBD3 binding to MER85 elements, we performed EMSAs with two strongly bound MER85 elements (MER85-360 and MER85-427, see Table S1) that contain the 13 bp TIR sequences required for transposition. Surprisingly, when the MER85s were cut in two at the unique DpnI site, only fragments containing the 5′-most 42 bp of MER85 sequence exhibited a mobility shift (Figure 3). We confirmed this result by EMSAs using synthetic 42-mers that corrected occasional mismatches between the two MER85s and the consensus MER85 sequence (Figure S1). Thus the TIR sequence is not sufficient for binding the PGBD3 transposase, and essential sequences of the transposase binding site must be located elsewhere within the 5′-most 42 bp of MER85 elements.

### An imperfect internal 16-bp palindrome is essential for binding of the CSB-PGBD3 fusion protein to the 5′ end of MER85 elements *in vitro*

**Figure 4:**
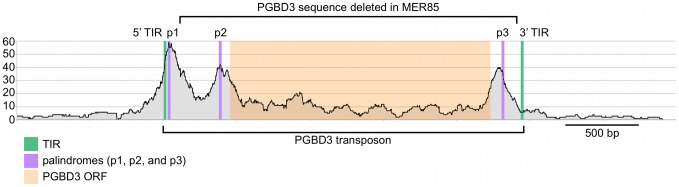
Fragment overlaps in the vicinity of the PGBD3 transposon reveal strong binding near each of three palindromes. Occupancy of CSB-PGBD3 near the PGBD3 transposon was assessed by counting the number of overlapping ChIPed fragments at each position. TIR, terminal inverted repeat; ORF, open reading frame. Ordinate indicates the number of overlapping fragments.

Visual inspection of MER85 sequences revealed an imperfect 16 bp palindrome GTTCCAtTAtTGGAAC located 3 bp internal to the 5′ TIR. The PGBD3 transposon that integrated into the CSB gene contains the same palindrome at three locations: once near the 5′ TIR as in MER85s, again 59 bp upstream of the PGBD3 transposase ORF, and yet again 75 bp downstream of the ORF termination codon and 114 bp upstream from the 3′ TIR (Figure 4; also see Figure S2 for conservation of the palindromes in PGBD3 pseudogenes). In MER85 elements, the sole palindrome lies 3 bp downstream from the 5′ TIR but 96 bp upstream of the 3′ TIR. Similar spacing between the 3′ most palindrome and the 3′ TIR in both the PGBD3 transposon (114 bp) and MER85s (96 bp) suggests that the sole MER85 palindrome may be functionally equivalent to the 3′ most palindrome in the full-length transposon, or may perhaps do double duty — functioning early in the reaction at the 5′ end and later at the 3′ end. A similar palindrome TGCGTaAAATTgACGCA, called the internal repeat, is found 3 bp downstream from the 5′ TIR and 31 bp upstream from the 3′ TIR of the piggyBac transposon from *Trichoplusia ni* [22]. A partial deletion of the 3′ internal repeat abolishes transposition [23], suggesting that the palindromes are functionally important for transposition by both the moth and human piggyBac elements.

**Figure 5:**
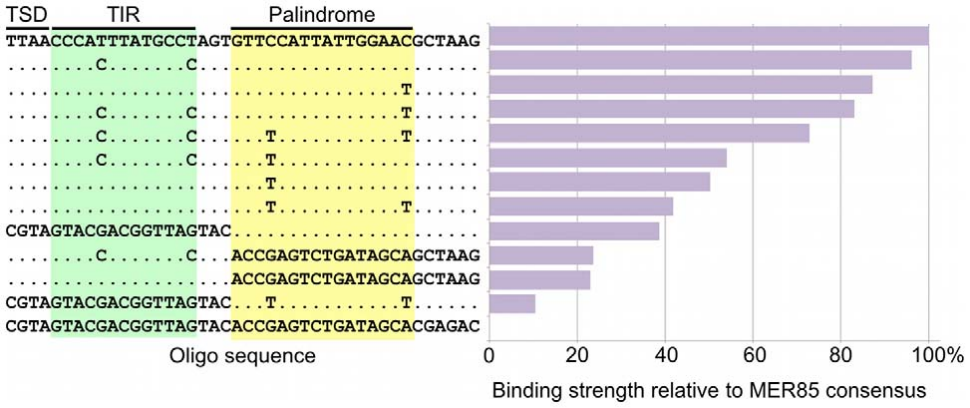
Mutations in the palindromic region reduce PGBD3 transposase binding affinity for MER85s. Synthetic 42 bp MER85 fragments were mixed with purified PGBD3 or no protein, and used for an electrophoretic mobility shift assay. The binding affinities of the transposase for synthetic 42 bp fragments were normalized to the Repbase consensus sequence (100%) and a scrambled sequence (0%). Only sequence mismatches are displayed; positions that match the Repbase consensus are indicated by periods. TSD, target site duplication; TIR, terminal inverted repeat.

To determine whether the 5′ palindrome of MER85s is required for PGBD3 binding, we examined MER85-65 in greater detail. This was the only MER85 in the panel of 6 that did not bind PGBD3 transposase *in vitro* or *in vivo*, despite being nearly identical in sequence to the other 5 elements [16]. Inspection of the 5′ end of MER85-65 revealed mismatches at 4 positions compared to the MER85 consensus: 2 in the TIR, and 2 in the palindrome (Figure 5). To test if the mutations in the 5′ TIR or the palindrome or both reduced the binding affinity, we performed EMSAs with 42 bp oligonucleotides that contained these mutations, singly and in combination, but otherwise matched the 5′ end of the MER85 consensus. Oligonucleotides with mutations that matched the MER85-65 TIR exhibited little loss of binding compared to the consensus. In contrast, oligonucleotides with mutations that matched the MER85-65 palindrome exhibited a 60% loss of binding (Figure 5), suggesting that mutations in the palindrome are likely responsible for the lack of binding to this particular element *in vivo*. No combination of mutations in the oligonucleotide gave as great a loss of binding as observed when the entire MER85-65 element was assayed by EMSA [16] or ChIP-seq (Figure 2), suggesting that other factors, such as sequence context or chromatin accessibility, may contribute to CSB-PGBD3 binding *in vivo*. To confirm the importance of the 5′ palindrome, we tested 42 bp oligonucleotides in which either the entire 5′ TIR or 5′ palindrome was replaced by random sequence. Surprisingly, deletion of either region reduced binding *in vitro*, but the effect was greater for the palindrome (80% loss) than for the TIR (60% loss) (Figure 5).

The fact that 5′ MER85 sequences favor DNA binding both *in vitro* (Figure 3) and *in vivo* (Figure 2, Table S1) suggests that the PGBD3 transposase alone is sufficient for initial recognition of the 5′ end of MER85 mobile elements. The ability of the moth element to function efficiently in mammalian cells further reinforces this interpretation [24]; however, host independence does not exclude the participation of auxiliary proteins that may facilitate or stabilize assembly of the transpososome [25].

### The CSB-PGBD3 fusion protein is enriched at >2,000 sites in the human genome

CSB-null UVSS1KO fibroblasts are derived from a patient with UV sensitive syndrome (UVSS) and express neither CSB [26] nor CSB-PGBD3 fusion protein [10] as a result of a homozygous nonsense mutation at CSB codon 77. We had previously generated gene expression array data for UVSS1KO cells stably expressing FLAG-HA-tagged CSB-PGBD3 fusion protein [16]. To correlate these expression array data with genome-wide CSB-PGBD3 chromatin binding profiles for the same cells, we used paired-end ChIP-seq [27] in which the cells are crosslinked with formaldehyde, sonicated, and sheared chromatin is immunoprecipitated with an antibody against the protein of interest — in this case a mouse monoclonal antibody against the N-terminal domain of human CSB. The immunoprecipitated DNA fragments are ligated to Illumina adapters, and 300–600 bp fragments are size-selected by PAGE and pre-amplified by PCR before loading onto the Illumina flow cell where one end of each captured fragment is sequenced. Synthesis of the opposite strand and cleavage of an 8-oxoguanine incorporated into the immobilized flow cell oligonucleotides then allow the fragments on the surface of the flow cell to be resequenced from the other end [27]. Paired-end sequencing greatly improves the mapping of repetitive DNA sequence elements such as MER85s because the short reads obtained from both ends of each sonicated chromatin fragment can be required to align uniquely with genomic sequences near each other and on opposite strands.

More than 8.5 million pairs of enriched ChIP-seq reads of 36 bp were mapped to human genome build hg18 (NCBI 36) using the read mapping program Bowtie [28]. Because CSB-PGBD3 binds to repetitive (and very similar) MER85 elements, we used stringent settings that disregard reads containing mismatches and reads that could not be uniquely mapped. The surviving reads were then analyzed for local enrichment using three independent peak-finding algorithms — Model-based Analysis of ChIP-Seq (MACS) [29], Enhanced Read Analysis of Gene Expression (ERANGE) [30], and Quantitative Enrichment of Sequence Tags (QuEST) [31] — which differ based on how the paired sequence tags are handled, as well as in the statistical methods used to determine peak enrichment (reviewed in [32]). Comparison of results from each algorithm allowed us to find peaks that were consistently enriched independent of the peak-calling method.

We found that 363 of 889 MER85 elements were reliably enriched and called as peaks by all 3 peak finding algorithms (Table S1). To prevent easily sheared chromatin regions and regions artefactually enriched by pre-amplification from scoring as peaks, each of our analyses included an input control consisting of ∼3 million single-end reads from the same sheared chromatin used for ChIP-seq. The 2,087 peaks found by all 3 algorithms were used for subsequent analysis (Table S2). We then wrote a Perl script to generate internally consistent CSB-PGBD3 binding profiles over all 2,087 peaks. The script converted mapped paired-end reads to the genomic coordinates of the corresponding ChIP fragments, calculated the number of fragments overlapping each position in the genome, and compiled the fragment map as a wiggle file to display and analyze CSB-PGBD3 binding profiles. A second script was used to locate the highest fragment overlap, defined as the peak summit, in each of the 2,087 enriched region identified by all three peak calling algorithms. We also used the Cis-regulatory Element Annotation System (CEAS) program [33] to show that CSB-PGBD3 peaks are significantly enriched within 3 kb of transcription start sites (6.1%, p-value 1.6e-20), although the vast majority of peaks are either intronic (41.6%) or in distal intergenic regions (47.8%) (Figure S3).

### The CSB-PGBD3 fusion protein binds to the 5′ end of 363 MER85 elements and the PGBD3 locus in CSB

We located all 889 MER85 elements in the hg18 build of the human genome, and examined them individually to ensure that the boundaries of each MER85 were correctly identified even in cases where expansions or insertions altered the length of MER85 elements. All MER85 elements are given in the same orientation as the parental PGBD3 transposon (Figure 1) with the 5′ and 3′ ends of the MER85s corresponding to the first ∼100 bp and last ∼40 bp of the transposon. Of these 889 MER85s, we found 813 with intact terminal inverted repeats (TIRs), 13 of which had large internal insertions or repeat expansions (>20 bp longer than normal) and 1 of which had an internal deletion. Of the remaining 76 MER85s, 22 had incomplete 5′ ends, 49 incomplete 3′ ends, and 5 lacked both TIRs (Table S1).

**Figure 6:**
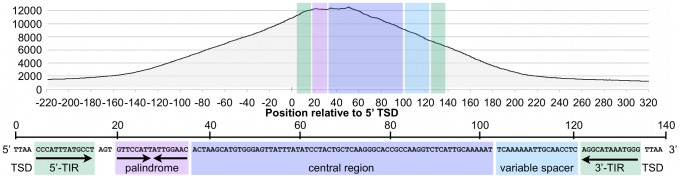
The CSB-PGBD3 fusion protein binds preferentially to the 5′ palindromic sequence of all bound MER85s in the human genome. Paired-end sequence reads near bound MER85s were used to reconstruct the location of immunoprecipitated fragments relative to the 5′ target site duplication (TSD) of each element. Cumulative fragment overlaps were calculated by summing the number of fragments from each element that overlapped each position relative to the 5′ TSD. TIR, Inverted Terminal Repeat.

When all bound MER85s were aligned in the same orientation, fragment overlaps indicated preferential binding to a 40 bp region just internal to the 5′ TIR (Figure 6), consistent with EMSA experiments on representative MER85 elements (Figure 3). No MER85 element lacking the 5′ palindrome bound CSB-PGBD3, although several elements that lacked 5′ or 3′ TIRs were reliably enriched (Table S1), further supporting our conclusion that CSB-PGBD3 binds primarily to the 5′ palindrome. Comparison of the 5′ palindrome sequences of bound and unbound MER85s revealed that 291 of 363 bound elements (80.1%) but only 48 of 526 unbound elements (9.1%) perfectly matched the consensus. The presence of unbound MER85s with perfect palindrome sequences suggests once again that other factors, such as chromatin accessibility, are likely to modulate CSB-PGBD3 binding *in vivo*. This could also explain why only 5 of the 6 MER85 elements used previously for EMSA correlated with the ChIP-PCR and ChIP-seq results (Figure 2).

We also examined binding of the CSB-PGBD3 fusion protein to the PGBD3 locus within the CSB gene, as well as to PGBD3 pseudogenes. Unexpectedly, the PGBD3 locus in CSB is one of the strongest and most extensive CSB-PGBD3 binding sites in the entire genome; moreover, paired-end fragment reads overlapped most heavily near each of three copies of the imperfect 16 bp palindromic sequence in the PGBD3 transposon (Figure 4). The same was true for the PGBD3 pseudogenes (Figure S2), but only where the palindromic repeats perfectly matched those of the full-length PGBD3 insertion in CSB (Figure S2). Although CSB is thought to be expressed in all tissues, and CSB mutations are recessive, it is unclear if or how binding of the CSB-PGBD3 fusion protein to the PGBD3 transposon affects CSB and/or PGBD3 transcription, splicing, or expression.

### TRE, TEAD1, and CTCF motifs are enriched in CSB-PGBD3 peaks

Much to our surprise, peaks over MER85, PGBD3, and PGBD3 pseudogenes accounted for only 367 (17.5%) of the 2,087 genomic regions enriched by immunoprecipitation with CSB-PGBD3. To determine what sequences in non-MER85 peaks were responsible for enrichment of CSB-PGBD3, we used MEME (Multiple Em for Motif Elicitation) to search for overrepresented sequence motifs located within 50 bp of non-MER85 peak summits [34]. Enriched motifs were then submitted to the TOMTOM motif comparison tool to identify known binding proteins [35].

**Figure 7:**
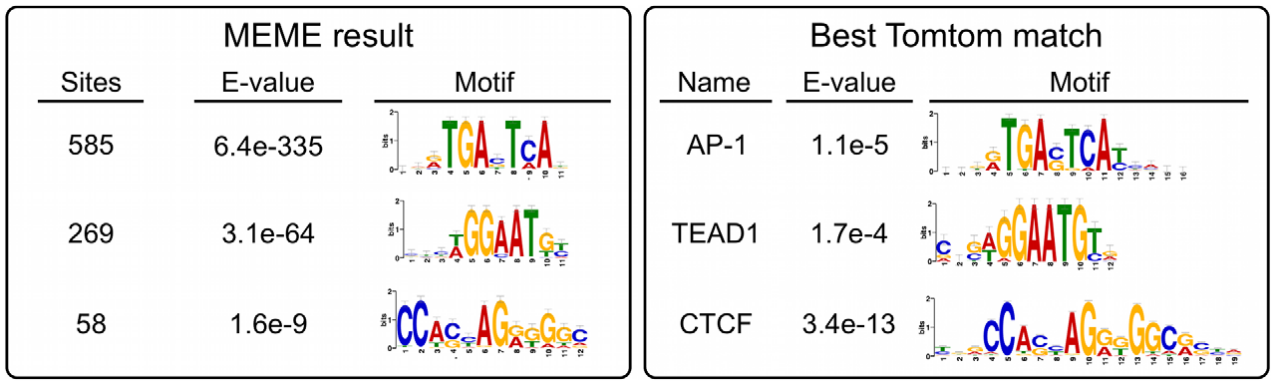
Non-MER85 peaks are enriched for TRE, TEAD1, and CTCF binding site motifs. (*left*) Analysis using Multiple Em for Motif Elicitation (MEME). Sequences within 50 bp of non-MER85 peak summits were submitted to MEME to identify overrepresented motifs. (*right*) Analysis using Tomtom motif comparison tool. Position specific frequency matrices for the motifs identified by MEME were submitted to TOMTOM to identify matching transcription factor binding sites. The most significant matches for each result are shown. *AP-1 motif was annotated jundm2_secondary, Jun dimerization protein 2 secondary motif (UniPROBE mouse database); TEAD1, TEA domain family member 1 (JASPAR core 2009 database); CTCF, CCCTC binding factor (JASPAR core 2009 database).

The top hit was the sequence TGANTCA found near 585 (28%) of the 2,087 peak summits (E-value = 6.4e-335) (Figure 7). This motif was identified by TOMTOM as the tumor promoting antigen response element (TRE) best known as the binding site for Activator Protein 1 (AP-1) family complexes [36].

The next most highly represented motif was [AT]GGAAT[GT] where [AT] is A or T, and [GT] is G or T; this motif is found near 269 (13%) of the 2,087 peak summits (E-value = 3.1e-64) and resembles the binding site for the TEAD1 (TEA domain family member 1) transcription enhancer protein (Figure 7). This motif is very similar to part of the MER85 palindromic region (TGGAACG), and we cannot entirely exclude the possibility that it is bound directly by CSB-PGBD3 because a C>T mutation within this motif (TGGAATG) only slightly reduced PGBD3 binding *in vitro* (Figure 5). On the other hand, 199 MER85 elements in the genome have this C>T mutation, yet only 6 are bound by CSB-PGBD3 in the ChIP-seq dataset (Table S1).

The third most significant motif with a known binding protein was CCA[CG][CT]AG[AG][GT]GGC, found near 58 (2.7%) of the 2,087 peak summits (E-value 1.6e-9) and was identified as the binding site for CTCF (CCCTC-binding factor), a key regulator of chromatin looping and other higher-order chromatin structures [37] (Figure 7).

**Figure 8:**
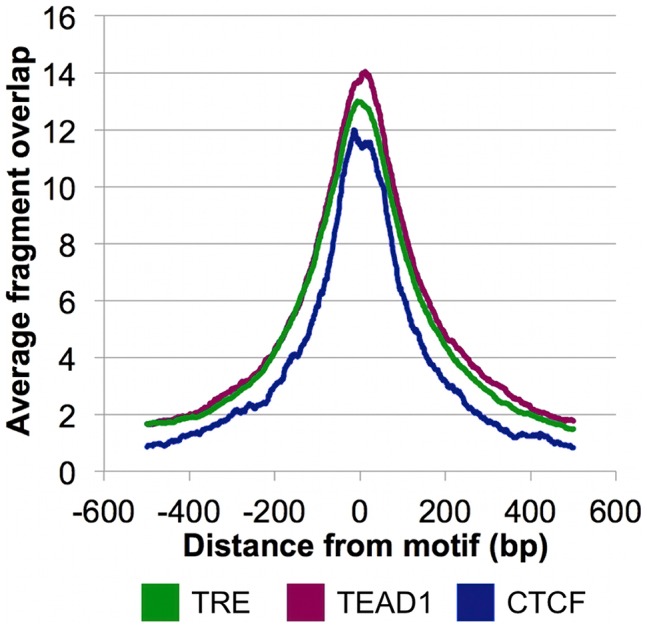
CSB-PGBD3 peak summits coincide with the TRE, TEAD1, and CTCF motifs. Average fragment overlaps in the vicinity of TRE, TEAD1, and CTCF motifs were plotted for the CSB-PGBD3 ChIP-seq data. The overlaps peak sharply and symmetrically around the motifs, consistent with tethering of the CSB-PGBD3 fusion protein to the corresponding transcription factors through protein-protein interactions.

The overrepresentation of these three motifs near the CSB-PGBD3 summits in non-MER85 peaks (tabulated in Table S3) suggests that CSB-PGBD3 may interact with all three of these DNA binding factors. Consistent with this interpretation, average fragment overlap profiles centered on these motifs show sharp accumulation of CSB-PGBD3 enriched fragments over the motifs (Figure 8). Alternatively, CSB-PGBD3 might bind directly to one or more of these motifs, for example through a cryptic activity of PGBD3 DNA binding domain.

### The AP-1 family protein c-Jun co-immunoprecipitates with the CSB-PGBD3 fusion protein

We used an EMSA assay to ask whether CSB-PGBD3 can bind directly to TRE motifs, or is more likely tethered to the motif by protein-protein interactions with TRE bindng factors. As anticipated, purified CSB-PGBD3 fusion protein failed to shift 42 bp oligonucleotides containing one or two TRE motifs, although control MER85 sequences shifted cleanly and random sequences did not shift at all (Figure S1).

To determine if binding of CSB-PGBD3 to TRE motifs is mediated by an interaction with a TRE binding protein, we asked whether CSB-PGBD3 would co-immunoprecipitate (coIP) with AP-1 proteins that are known to bind TRE motifs. AP-1 complexes are composed of many homo- or heterodimeric combinations of members of the Jun, Fos, Maf, and ATF protein families, and the combination of AP-1 family members determines the affinity of the complex for specific variants of the sequence motifs [19], [38]. Fos and Jun bind preferentially to the TRE sites (TGANTCA) identified in CSB-PGBD3 peaks, and more weakly to the similar cyclic AMP response element binding site (TGACGTCA). Although the binding repertoire of Jun and Fos can be expanded through interactions with several other DNA binding proteins [39], the CSB-PGBD3 peaks contain only TRE motifs suggesting that CSB-PGBD3 interacts directly with Jun or Fos proteins.

**Figure 9:**
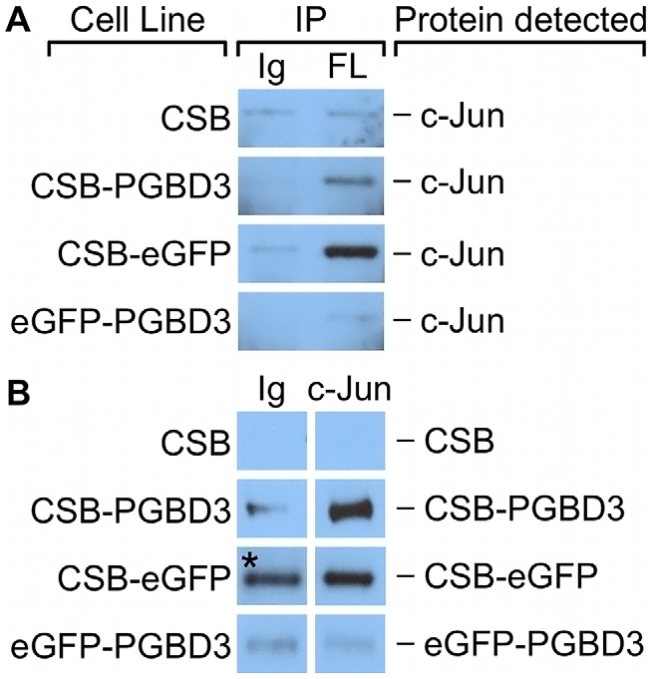
c-Jun co-immunoprecipitates with the CSB-PGBD3 and CSB-eGFP proteins, but not with eGFP-PGBD3. Nuclear lysates from UVSS1KO cells stably expressing FLAG-HA-tagged CSB, CSB-PGBD3, CSB-eGFP, and eGFP-PGBD3 were immunoprecipitated using anti-FLAG, anti-c-Jun, and a nonspecific antibody. (A) Western blots probed with anti-c-Jun antibodies. c-Jun is immunoprecipitates with anti-FLAG antibodies in cells expressing FLAG-HA-tagged CSB-PGBD3 or CSB-eGFP, but not full-length CSB or eGFP-PGBD3. (B) Western blots probed with anti-FLAG antibodies. FLAG-HA-tagged CSB-PGBD3 and CSB-eGFP immunoprecipitate with anti-c-Jun antibodies. * Denotes lane with uncharacteristically high background. The same nonspecific antibody was used for all negative control samples, which leads us to believe this band is an artefact due to contamination rather than a true IP of CSB-eGFP. IP, antibodies used for immunoprecipitation; Ig, anti-mouse IgG nonspecific antibody control; FL, mouse monoclonal anti-FLAG antibody; c-Jun, anti-c-Jun antibody.

The Jun and Fos genes c-Jun, JunD, Fra1, and Fra2 have previously been shown to be expressed in exponentially growing fibroblast cultures [40]. We were able to detect expression of Jun, JunD, and Fra2 in our UVSS1KO-derived fibroblast lines by Western blotting (Figure S4) but not Fra1 (data not shown). In UVSS1KO cells stably expressing FLAG-HA-tagged CSB-PGBD3, coIPs with antibodies against c-Jun enriched for CSB-PGBD3 compared to a non-specific antibody control (Figure 9B) but coIPs with antibodies against JunD and Fra2 did not (data not shown). Moreover, reciprocal coIPs with anti-FLAG antibodies enriched for c-Jun in cells expressing FLAG-HA-CSB-PGBD3 (Figure 9A). These results suggest that CSB-PGBD3 binds to TRE sites indirectly, through a protein-protein interaction with bound c-Jun.

To localize the site of interaction on CSB-PGBD3, we repeated the coIPs in cells expressing FLAG-HA-tagged chimeric CSB-eGFP, eGFP-PGBD3, or full-length CSB (Figure 9B). Of these cell lines, only CSB-eGFP enriched for c-Jun in an anti-FLAG coIP. Thus c-Jun interacts with the N-terminus of CSB in the CSB-PGBD3 fusion protein, but not with the N-terminus of intact CSB protein. CSB may fail to bind c-Jun because the autoinhibitory N-terminal domain preferentially interacts with the C-terminal helicase domain in the intact protein [13].

### CSB-PGBD3 binding to TRE motifs, but not MER85s, correlates with regulation of nearby genes in CSB-null UVSS1KO cells and with CSB repression in CS1AN cells that continue to express the CSB-PGBD3 protein

We used the Genomic Regions Enrichment of Annotations Tool (GREAT) [41] to ask whether genes that are regulated by the CSB-PGBD3 fusion protein [16] are located near CSB-PGBD3 binding sites as determined by ChIP-seq. We previously generated expression array datasets for stable expression of CSB-PGBD3, CSB, both proteins, or neither in CSB-null UVSS1KO cells [16] but these data had not yet been entered into a database used by the online version of GREAT. Instead, we used a local copy of the GREAT tool, Calculate Binomial P-Value, to correlate our CSB-PGBD3 expression array and ChIP-seq data. We also compared our CSB-PGBD3 ChIP-seq data to genes up- and downregulated when the CS1AN cell line, a patient-derived CSB compound heterozygote, was rescued with wild-type CSB [9].

GREAT tests for statistical enrichment of peaks in regions near a set of genes. To do this, GREAT defines “regulatory domains” that extend in both directions for a specified distance from the transcription start site (TSS) or to the next nearest gene. Using regulatory domains of 100 kb, 250 kb, and 1 Mb, we tested sets of genes that were up- and downregulated under each condition separately, and compared them to the set of all 2,087 CSB-PGBD3 peaks. We also used GREAT to correlate our expression array datasets with CSB-PGBD3 peaks over MER85 elements (363 peaks), TRE motifs (585 peaks), TEAD1 motifs (269 peaks), CTCF motifs (58 peaks), and peaks that contain none of these motifs (892). Very few peaks contained more than one motif except for 72 peaks with both TRE and TEAD1 motifs, and for consistency these TRE+TEAD1 peaks were counted as members of both peak sets. For each comparison, 100 sets of randomized peak locations were used as negative controls and to calculate empirical false discovery rates (FDR) [42]. Only comparisons with an FDR of less than 1% were considered significant (Table S4).

GREAT analysis revealed that peaks containing TRE motifs are significantly enriched near genes upregulated and downregulated by CSB-PGBD3 using all of the regulatory domain sizes (orange cells in Table S4). Enrichment of TRE motifs near upregulated and downregulated genes suggests that CSB-PGBD3 interacts with AP-1 proteins to modulate the expression of nearby genes. In contrast, peaks over MER85 elements did not correlate significantly with any of the UVSS1KO or CS1AN expression array datasets (gray cells in Table S4), despite enrichment of MER85 elements near specific gene ontology (GO) categories [10]. This suggests that regulation of gene expression by the CSB-PGBD3 fusion protein is strongly dependent on location and cooperation with other transcription factors; simple DNA binding in the vicinity of genes is not sufficient. These results support a very different model from our initial speculation that CSB-PGBD3 binding would create a MER85-based transcriptional network. Instead, it appears that CSB-PGBD3 selectively interacts with existing transcription factors to provide an additional layer of gene regulation on top of established regulatory networks.

### CSB-PGBD3 and CSB may coregulate expression of specific genes in normal individuals

In addition to analysis of genes regulated by CSB-PGBD3 expression alone, we also compared CSB-PGBD3 binding to genes regulated by coexpression of CSB and CSB-PGBD3 in the same CSB-null cell line UVSS1KO. Importantly, this set of genes is distinct from genes regulated by CSB or CSB-PGBD3 alone, suggesting that co-regulation could be the result of direct interactions between the N-terminus of CSB-PGBD3 and CSB [13] or indirect interactions in which upregulation of certain genes by CSB-PGBD3 requires prior (or concurrent) chromatin remodeling by CSB [9]. GREAT analysis revealed that many genes which are upregulated by coexpression of CSB and CSB-PGBD3, but not by either protein alone, correlate significantly with the set of all peaks bound by CSB-PGBD3 and with the subsets of peaks over TRE and TEAD1 motifs (blue cells in Table S4). These genes could in principle be regulated by the N-terminal domain of CSB, CSB-PGBD3, or both; however, we might then have expected to see a similar correlation with genes upregulated by stable expression of CSB alone. It therefore seems more likely that this subset of genes is upregulated by CSB-PGBD3 through interactions that are enhanced by or require CSB, and thus may also be upregulated in normal, healthy individuals.

In contrast, many genes that are upregulated by expression of CSB-PGBD3 in CSB-null cells are repressed by coexpression of CSB [16]. Moreover, 16 of these genes are also downregulated (binomial p-value 6e-7) when CSB is expressed in CS1AN cells that continue to express the CSB-PGBD3 fusion protein despite loss of functional CSB [10]. Of these 16 genes, 8 have CSB-PGBD3 binding sites within 100 kb of the TSS (ARHGAP29, IGFBP7, MGLL, PODXL, PSG1, RGMB, RGS4, and SERPINE1) suggesting that CSB can repress some, but not all genes that are upregulated by nearby CSB-PGBD3 fusion protein — perhaps depending on local context or the specific transcription factor(s) that tether CSB-PGBD3 to the site.

### CSB-PGBD3 peaks correlate with diverse ontologies related to angiogenesis, the TGF-beta pathway, cancer, and immune responses

Our expression array analysis was limited to several cell lines and culture conditions. To investigate the role of CSB-PGBD3 binding sites in the broader context of human biology and disease, we used the online version of GREAT to compare our binding sites to a diverse set of gene ontologies. Using the default settings, we submitted either the full set of CSB-PGBD3 peaks to GREAT, or the subsets containing the MER85, TRE, TEAD1, or CTCF motifs, or no recognizable motif. The MER85 and CTCF (as well as TRE+TEAD1) peaks did not exhibit statistically significant overlaps with any ontology sets, but for the other peak categories we examined the top five results in the GO Biological Processes, Disease Ontology, Pathway Commons, and MSigDB Perturbation datasets (Table S5). We found that CSB-PGBD3 binding sites correlated significantly with genes related to the TGF-beta pathway, carcinogenesis, and IFN and IL-2 driven innate immune responses (see Table S5 legend for details).

### CSB-PGBD3-bound TRE motifs are enriched near CSB-PGBD3-bound MER85 elements

MER85 elements are among the strongest CSB-PGBD3 binding sites *in vivo*, yet bound MER85 elements do not correlate with genes induced or repressed by CSB-PGBD3 expression in CSB-null UVSS1KO cells (Table S4). Thus we must consider the possibility that continued binding of the CSB-PGBD3 fusion protein to MER85s might be fortuitous or functionless. The burst of MER85 replication apparently came to an end about 35 Mya [43], perhaps upon mutation of the conserved catalytic aspartate (D352) in the PGBD3 transposase ORF to asparagine [10], [44]. The limited sequence diversity of the surviving 889 human MER85s (Table S1), the ability of the CSB-PGBD3 binding site to tolerate point mutations and even deletions (Figure 5), and the small target size of the essential 16 bp imperfect palindrome (Figure 5 and Figure 6), are all consistent with our observation that at least 40% (363/889) of all MER85s retain the ability to bind the PGBD3 transposase (Table S1) despite ongoing mutations over the past 35 My. We conclude that neutral sequence evolution could have been sufficient to account for the homogeneity and current functions of MER85 elements.

Alternatively, binding of the CSB-PGBD3 fusion protein to MER85s through the PGBD3 domain may enable CSB-PGBD3-mediated chromosome looping with transcription factors bound to TRE, TEAD1, or CTCF motifs. To test this hypothesis, we used the GREAT tools to determine if CSB-PGBD3 binding sites containing TRE, TEAD1, or CTCF motifs (Table S3) were significantly enriched within 100 kb of MER85s that are bound by CSB-PGBD3 (Table S1). Surprisingly, we found a strong correlation between CSB-PGBD3 peaks containing TRE motifs and the 363 MER85 elements bound by CSB-PGBD3 (36 of 585 bound TRE motifs, P-value = 7.9e-7) but not with the 529 unbound MER85 elements (16 of the 585 bound TRE motifs, P-value = 0.88). Peaks containing TEAD1 or CTCF motifs, and peaks containing no identified motif, showed no enrichment near bound or unbound MER85 elements.

### CSB-PGBD3 interacts with RNAPII

**Figure 10:**
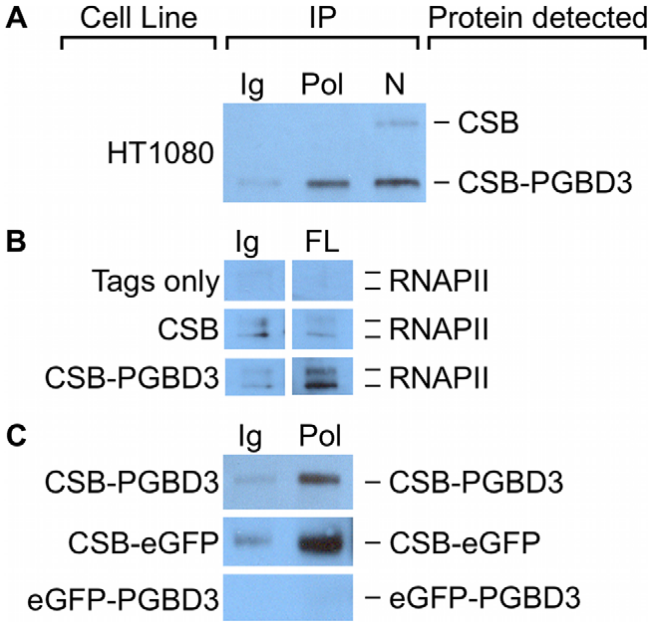
CSB-PGBD3 and CSB-eGFP co-immunoprecipitate with RNA polymerase II (RNAPII). (A) HT1080 whole cell lysates were immunoprecipitated using anti-RNAPII CTD antibodies, N-terminal CSB antibodies, or nonspecific antibodies. CSB and CSB-PGBD3 were detected by western blotting with antibodies against the N-terminus of CSB. (B) UVSS1KO cells expressing FLAG-HA tags only, FLAG-HA-tagged CSB, or FLAG-HA-tagged CSB-PGBD3 were immunoprecipitated using antibodies for FLAG tags or a nonspecific antibody control. RNAPII was detected by western blotting with antibodies against the CTD of RNAPII. (C) UVSS1KO cells expressing FLAG-HA-tagged CSB-PGBD3, CSB-eGFP, or eGFP-PGBD3 were immunoprecipitated with antibodies against the CTD of RNAPII or a nonspecific antibody control. CSB-PGBD3, CSB-eGFP, and eGFP-PGBD3 were detected by western blotting with anti-FLAG antibodies. Ig, anti-mouse IgG nonspecific control; Pol, anti-RNAPII CTD; N, anti-CSB N-terminus; FL, anti-FLAG.

CSB interacts with stalled RNAPII after induction of DNA damage [6], but it also copurifies with RNA polymerase II in unirradiated cells [4] and thus may associate with transcribing RNA polymerase II (RNAPII) as well as with TCR complexes. To determine whether some of the genomic CSB-PGBD3 peaks might reflect interaction of CSB-PGBD3 with RNAPII, we asked if antibody against the C-terminal domain (CTD) of RNAPII could co-immunoprecipitate (coIP) CSB-PGBD3, and vice versa. Intriguingly, coIPs with antibody against the RNAPII C-terminal domain enriched for CSB-PGBD3 but not CSB in undamaged HT1080 human fibrosarcoma cells (Figure 10a). In a reciprocal coIP using UVSS1KO cells that stably express FLAG-HA-CSB, FLAG-HA-CSB-PGBD3, or the FLAG-HA tags only, coIPs with anti-FLAG antibody enriched for RNAPII in cells expressing CSB-PGBD3, but not in cells expressing intact CSB or tags only (Figure 10b). The failure of CSB to co-immunoprecipitate RNAPII is more likely to reflect low affinity between CSB and RNAPII in the absence of DNA damage than accessibility of the tags, because anti-FLAG IPs readily pull down FLAG-HA-CSB in UVSS1KO cells (data not shown).

To see if interactions between CSB-PGBD3 and RNAPII could account for CSB-PGBD3 peaks that did not contain an overrepresented sequence motif, we compared regions within 50 bp of CSB-PGBD3 peaks to enriched RNAPII peaks obtained from the Yale TFBS collection in the UCSC Genome Browser database [45], [46]. Because RNAPII binding sites vary between cell types, we analyzed 18 RNAPII genome-wide peak sets from 15 cell lines. We found 105 of 2087 CSB-PGBD3 peaks consistently overlapped at least 10 of 18 RNAPII peak sets (Table S6), and that 85 of these CSB-PGBD3 peaks did not contain a MER85, TRE, TEAD1, or CTCF motif. The set of 105 CSB-PGBD3 peaks that overlapped RNAPII peaks were compared to expression array datasets using GREAT as described previously. Peaks associated with RNAPII binding sites were enriched near genes upregulated by coexpression of CSB and CSB-PGBD3, but not by expression of either protein alone (Table S4). Thus, interactions between CSB-PGBD3 and RNAPII may require regulation or remodeling of the gene by CSB [9].

We localized the region of interaction between CSB-PGBD3 and RNAPII by asking whether antibody against the CTD of RNAPII would immunoprecipitate stably expressed FLAG-HA-CSB-eGFP, FLAG-HA-eGFP-PGBD3, or FLAG-HA-CSB-PGBD3 from UVSS1KO cells. Surprisingly, RNAPII interacts with CSB-eGFP but not with eGFP-PGBD3 (Figure 10c). Thus, CSB-PGBD3 interacts with RNAPII through the N-terminal CSB domain, just as it does with c-Jun (Figure 9). The implication may be that the highly conserved SWI/SNF ATPase domain encoded by CSB exons 6–21, although unlikely to be a generic chromatin remodeler [47], is modulated, autoinhibited [13], and targeted to specific chromosomal locations by the N-terminal domain (coding exons 2–5) (Figure 10 and Figure S1).

### The N-terminal CSB and C-terminal PGBD3 domains of CSB-PGBD3 can independently alter gene expression

The ability of the N-terminal domain of CSB to interact directly with c-Jun (Figure 9) and RNAPII (Figure 10), as well as the failure of CSB-PGBD3 fusion protein to affect expression of nearby genes when bound to MER85s (Table S4), suggested that CSB-PGBD3 could potentially regulate gene expression without binding directly to DNA. To test if the CSB N-terminus alone can induce the changes in gene expression caused by CSB-PGBD3, we stably expressed two chimeric fusion proteins in UVSS1KO cells: a CSB-LacI chimera in which the C-terminal PGBD3 domain is replaced by LacI, and the reciprocal eGFP-PGBD3 chimera in which the N-terminal CSB domain is replaced by eGFP. Using quantitative PCR (QPCR), we then compared the relative expression of selected genes in the stable lines expressing the CSB-LacI, eGFP-PGBD3, and control CSB-PGBD3 constructs. We selected a panel of 23 genes for the QPCR assay: 13 genes that were upregulated (signal log ratio SLR>1) when CSB-PGBD3 was stably expressed in the CSB-null UVSS1KO line [16], 7 genes that were downregulated (SLR<−1), and 3 genes that showed no significant change in expression (SLR between 1 and −1).

**Figure 11:**
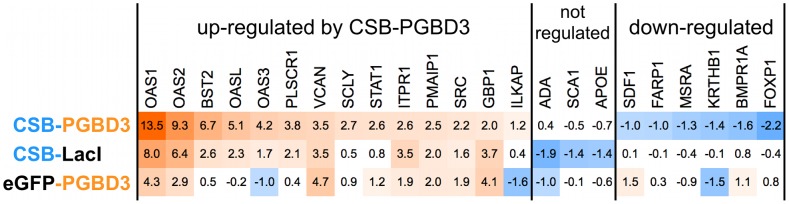
CSB-LacI and eGFP-PGBD3 induce partial up-regulation of genes regulated by CSB-PGBD3. Average signal log ratios from quantitative PCR (QPCR) of genes regulated by CSB-PGBD3, CSB-eGFP, or eGFP-PGBD3 expression in UVSS1KO cells compared to cells expressing FLAG-HA-tags alone. Orange cells: increased expression; Blue cells: decreased expression; No color: expression change was less than 2-fold (1 signal log ratio); darker color indicates a larger change in expression.

Most of the 23 genes exhibited similar expression changes in both expression array and QPCR experiments: 14 genes were upregulated at least 2-fold (SLR>1) by CSB-PGBD3, 6 genes downregulated at least 2-fold (SLR<−1), and 3 genes exhibited less than 2-fold changes in expression by QPCR (Figure 11). The two exceptions were the v-src sarcoma viral oncogene homolog (SRC) which appears elevated by QPCR but not by microarray, and the spinocerebellar ataxia 1 gene (SCA1 or ataxin 1) which appeared to be less downregulated in the QPCR than in the microarray assay (SLR of −0.5 and −1, respectively). Thus the QPCR assays are consistent with our earlier expression array analysis [16].

Of the 14 genes upregulated by CSB-PGBD3, 11 were also upregulated by CSB-LacI, although less so for 8 of the 11 (Figure 11). Similarly, 8 of the same 14 genes were upregulated by eGFP-PGBD3, although less so for 6 of the 8 genes (Figure 11). These data suggest that both the N-terminal CSB domain and the C-terminal PGBD3 domain can independently upregulate genes induced by the CSB-PGBD3 fusion protein, but less effectively than when tethered together in a single protein. In contrast, the 6 genes downregulated by CSB-PGBD3 were almost unchanged by expression of CSB-LacI or eGFP-PGBD3 (Figure 11). CSB-LacI failed to downregulate any of these 6 genes by as much as the 2-fold cutoff for significance, and eGFP-PGBD3 downregulated only 1 of the 6 (Figure 11). Thus, downregulation of genes by CSB-PGBD3 requires fusion of the N- and C-terminal domains. Neither the CSB N-terminus nor C-terminal PGBD3 domain alone is capable of fully recreating the expression changes induced by CSB-PGBD3, and fusion of the two domains results in a transcriptional response that is greater than and somewhat different from the effect of the two domains individually. This could explain why the CSB-PGBD3 fusion has been conserved despite the presence of the CSB N-terminus in intact CSB and the intact PGBD3 protein transcribed from the cryptic promoter in CSB exon 5 (Figure 1 and [10]).

## Discussion

We have previously shown that expression of the CSB-PGBD3 fusion protein upregulates many genes related to innate immunity and an interferon-like antiviral response [16]. We also found that the PGBD3 domain of CSB-PGBD3 can bind MER85 elements *in vitro*, and therefore speculated that the CSB-PGBD3 fusion protein might regulate expression of nearby genes by binding to MER85 elements *in vivo*. Such binding could in principle affect gene expression in any of several ways: CSB-PGBD3 binding near many IFN-related genes or a few master regulators of the IFN response could drive an innate immune response directly. Alternatively, the N-terminal CSB domain of the CSB-PGBD3 fusion protein might act as a dominant negative in the absence of functional CSB, interfering with chromatin remodeling, and perhaps generating double-stranded RNA through bidirectional transcription, thus triggering innate immunity by mimicking a viral infection. And lastly, CSB-PGBD3 might affect gene expression not by binding site-specifically to MER85s but by interacting with unbound nucleoplasmic proteins. To begin to assess these models for regulation of gene expression by the CSB-PGBD3 fusion protein, we determined the genome-wide binding patterns of CSB-PGBD3, and used the Genomic Regions Enrichment of Annotations Tool (GREAT) to explore correlations between CSB-PGBD3 binding and gene regulation. As is often the case, the results were more interesting than the hypotheses.

**Figure 12:**
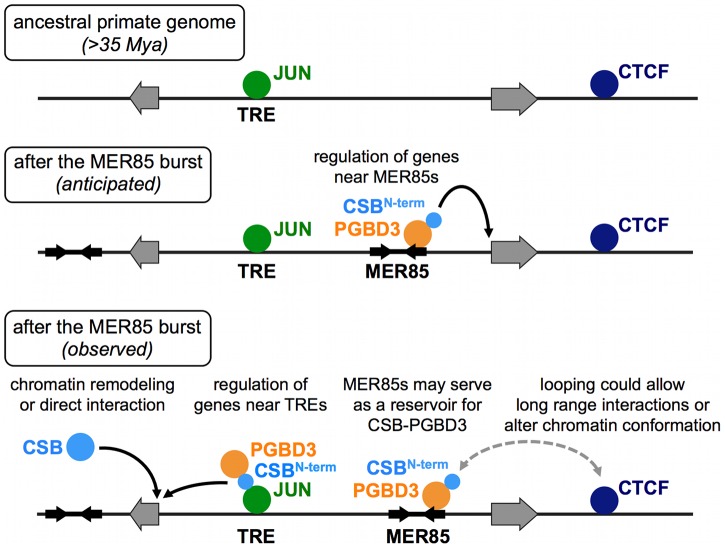
ChIP–seq data suggest multiple roles for the CSB-PGBD3 fusion protein in gene regulation. *Top*, Transcription factor binding sites before the MER85 replicative burst. *Middle*, We anticipated that the CSB-PGBD3 fusion protein would bind to MER85 elements throughout the genome and regulate nearby genes through interactions mediated by the N-terminal CSB domain. *Bottom*, Our ChIP-seq data revealed that CSB-PGBD3 binds over TRE, CTCF, and TEAD motifs, and regulates genes near TRE motifs in CSB-null cells. Full-length CSB may facilitate or suppress these interactions through chromatin remodeling or competition for factors that also bind the N-terminal CSB domain of CSB-PGBD3. The CSB-PGBD3 fusion protein does bind to MER85 elements as anticipated, but these sites may function as a reservoir for CSB-PGBD3 protein or mediate chromatin looping, perhaps by interaction with CTCF.

Our genome-wide analysis of CSB-PGBD3 binding sites and related experiments have demonstrated that (1) CSB-PGBD3 is recruited not only to MER85 elements and MER85-related sequences within the PGBD3 transposon, but also to TRE, TEAD1, and CTCF motifs throughout the genome, as well as to sites of RNAPII enrichment in diverse cell lines; (2) binding of CSB-PGBD3 fusion protein to TRE motifs, but not to MER85s, correlates with genes upregulated by expression of CSB-PGBD3 in CSB-null UVSS1KO cells and genes downregulated by expression of functional CSB in CS1AN cells; (3) the CSB-PGBD3 fusion protein interacts with the TRE binding transcription factor c-Jun and RNAPII through the N-terminal CSB domain; (4) full regulation of genes by CSB-PGBD3 requires fusion of the CSB and PGBD3 domains; and (5) TRE motifs that bind CSB-PGBD3 are significantly enriched near bound but not unbound MER85 elements. These results suggest a far more complicated model for domestication of the CSB-PGBD3 fusion protein than we had originally anticipated (Figure 12). The CSB-PGBD3 fusion protein does indeed bind to MER85 elements throughout the genome as hypothesized, but these sites do not appear to correlate with regulation of nearby genes; instead, gene regulation reflects binding of CSB-PGBD3 to existing chromatin-bound transcription factors and, quite possibly, to RNAPII as well.

### A new layer of regulation on established regulatory networks

Genome-wide binding of transposase-derived transcription factors had been demonstrated in Arabidopsis [48], but we provide a first look at the genome-wide binding of a transposase in transition: the PGBD3 transposase still binds strongly to related transposons, but has acquired novel functions because fusion with the N-terminus of CSB enables it to interact with previously established transcription factor networks. We had initially expected that binding of CSB-PGBD3 to MER85 elements would correlate with gene regulation induced by CSB-PGBD3. However, we found that CSB-PGBD3 interacts with a much broader range of binding sites, including TRE, TEAD1, and CTCF motifs, each of which is bound by factors that long predate horizontal transfer of PGBD3 to primate genomes. These results suggest that the conservation of the CSB-PGBD3 fusion protein over 43 My is due at least in part to modulation of existing regulatory networks rather than the creation of a *de novo* network based on insertion of MER85 elements near genes. However, the CSB-PGBD3 fusion protein also continues to bind MER85 elements, so we cannot rule out scenarios in which the fusion protein, bound to MER85 elements, regulates expression of nearby genes in specific cell types that we have not tested, or in occasional instances that would not appear statistically significant in our GREAT analysis. Thus a new protein (or RNA) that can modify established regulatory circuits may be able to build new functions without disrupting the old, whereas a new protein or regulatory RNA that can generate regulatory circuits de novo may be too powerful to survive because it would more likely do harm than good.

Nonetheless, transposable elements can, under very special circumstances, create regulatory networks *de novo*. For example, placental mammals express a large network of genes driven by transcription factor binding sites in MER20 transposons that regulate differentiation of endometrial stromal cells required for embryo implantation [49]. MER20s are present in >16,562 copies per human genome, 42% of which are located within 200 kb of the transcription start sites for pregnancy-induced genes. Moreover, the 218 bp element contains at least 22 potential binding sites for a total of 10 transcription factors (YY1, p300, C/EBPβ, CTCF, TGIF, p53, HoxA-11, FOXO1A, ETS1, and PGR), and quite remarkably 5 of these (C/EBPβ, PGR20, PGR21, FOXO1A, and HoxA11) are known to be important for hormone responsiveness and endometrial expression during pregnancy. The ability of MER20 to introduce a cluster of functional — and functionally related — transcription factor binding sites in a single insertional event may account for the evolutionary success of the MER20-based transcriptional network; it seems almost inconceivable that nearly identical clusters could have arisen at multiple genomic locations by neutral, stepwise mutation [50]. MER85s (some with bound CSB-PGBD3 fusion protein) could in principle participate in similar regulatory networks, as many MER85 elements contain binding sites for FOXA2, GFI, HAND1, HMGIY, HNF1A, NFE2L1, RORA, SOX5, and SRF (see Figure S5 for potential MER85 transcription factor binding sites), but this seems less likely because MER85s are 20-fold less abundant than MER20s (889 versus 16,562 copies per genome).

### CTCF and CSB-PGBD3 may play roles in chromosomal looping

CSB-PGBD3 may also regulate genes by affecting higher-order chromatin structure and looping. Our MEME analysis revealed the distinct signature of the CTCF binding motif in 58 CSB-PGBD3 peaks (Table S3, Figure 7). CTCF acts as a transcription activator or repressor depending on context, as a defining factor for gene insulation and silencing, and as a master regulator of long-range chromatin looping [37]. Although CTCF peaks represent only a small fraction of all CSB-PGBD3 binding sites, these peaks suggest that CSB-PGBD3 interacts directly with CTCF, perhaps mediating long-range interactions with CSB-PGBD3 bound to MER85s, TRE and TEAD1 motifs, or sites enriched for RNAPII. An interaction between CSB-PGBD3 and CTCF, through either the N-terminal CSB or C-terminal PGBD3 transposase domain, might also facilitate transposition. Intriguingly, the CTCF binding network in mammals has been shaped in part by retroposition of SINE elements that contain a CTCF motif [50], further expanding the repertoire of mechanisms by which transposons affect the structure and function of eukaryotic genomes.

CSB-PGBD3 could even play a direct role in chromosome looping (Figure 12). We found, by comparing all subsets of peaks in the ChIP-seq dataset for the CSB-PGBD3 fusion protein, that TRE motifs bound by the fusion protein are significantly enriched within 100 kb of MER85 elements that are also bound by fusion (see Results for details). Although it is possible that the fusion protein binds to these pairs of peaks independently, the data are consistent with chromosome looping mediated by the bifunctional fusion protein: the C-terminal PGBD3 transposase domain would bind to the MER85 and the N-terminal CSB domain would bind to AP-1 family transcription factors bound to the TRE motif — thus linking two distant sites, both of which would generate peaks in the ChIP-seq experiment.

### Additional roles for the N-terminal domain of CSB

The full-length CSB protein plays an essential role in TC-NER by recognizing stalled RNAPII and initiating assembly of the large TC-NER complex [5]–[7]. As these interactions had not yet been mapped to specific domains of CSB, we were surprised to find that both the CSB-PGBD3 fusion protein and the chimeric CSB-eGFP protein are able to interact with RNAPII (Figure 10) although these proteins contain only the N-terminal domain of CSB and none of the 7 conserved ATPase motifs (Figure 1 and [16]). We do not yet know whether the interaction between RNAPII and the N-terminal domain of CSB occurs on DNA or at sites of stalled RNAPII, but our co-immunoprecipitation experiments demonstrate that CSB and CSB-PGBD3 can share protein interaction partners through the common N-terminus. In fact, competition between CSB and CSB-PGBD3 for binding partners could play a role in CSB-dependent processes because expression of CSB-PGBD3 is about 4-fold higher than CSB in all cell lines we have examined [10].

The N-terminal domain of CSB has been shown to autoinhibit both normal and UV-induced association of CSB with chromatin [13], [51], but deletion of the N-terminal acidic tract had no obvious effect on repair of UV damage [11], [12]. Our data suggests that the N-terminus of CSB may play a larger role in targeting CSB to specific genes or chromosomal regions. We were surprised to find that the N-terminal domain of the CSB-PGBD3 fusion protein interacts both with both RNAPII (Figure 10) and c-Jun (Figure 9), and the sharp fragment accumulation profiles over TEAD1 and CTCF motifs (Figure 8) suggest that the fusion protein may also interact directly with TEAD1 and CTCF transcription factors bound to DNA. It also seems likely that the N-terminus of the CSB-PGBD3 fusion protein is responsible for binding to at least some of the 892 CSB-PGBD3 peaks (43% of 2,087 peaks total) that have no currently identifiable sequence motifs, but very likely bind transcription factors, chromosomal proteins, or enzymes such as topoisomerase I [15] involved in RNA and DNA transactions.

### A transcriptional role for CSB-PGBD3 in UV repair?

UV irradiation and other stressors activate c-Jun through phosphorylation by c-Jun N-terminal kinases (JNKs, also called stress-activated kinases) such as JNK1 [52]. Activated JNK and AP-1 complexes can then affect cell proliferation and apoptosis, depending on cell type and stimulus [36], [53]. We have previously shown that the CSB-PGBD3 fusion protein, although lacking all 7 ATPase motifs, can partially rescue UV damage repair in a host-cell reactivation assay using CSB-null UVSS1KO cells [16]. Conceivably, CSB-PGBD3 may facilitate repair by interacting with TC-NER proteins that normally associate with full-length CSB. However, the interaction between the CSB-PGBD3 fusion protein and the AP-1 family protein c-Jun (Figure 7, Figure 9, Figure S1) near genes upregulated by CSB-PGBD3 expression (Table S4) suggests an alternative scenario in which CSB-PGBD3 plays a transcriptional role in repair. After UV damage, activated AP-1 complexes could help guide CSB-PGBD3 to genes that are activated in response to UV. CSB-PGBD3 might then recruit RNAPII to these UV-activated TREs if the interactions of the N-terminal CSB domain of CSB-PGBD3 with c-Jun (Figure 7, Figure 9, Figure S1) and RNAPII (Figure 10) are not mutually exclusive.

### Does the CSB-PGBD3 fusion protein regulate CSB expression?

We were surprised to find that PGBD3 is strongly bound by CSB-PGBD3 near three palindromic motifs that are also present in the 5′ end of bound MER85s (Figure 4). Binding to these palindromes may autoregulate CSB transcription, CSB-PGBD3 expression, or alternative splicing and polyadenylation — perhaps by modulating the rate of RNAPII transcription or through interactions between the acidic N-terminus of CSB and phosphorylated serine/arginine-rich motifs in SR-family splicing enhancer proteins [54]. Thus it is possible that the CSB-PGBD3 fusion protein was initially retained in order to regulate CSB expression, and only secondarily acquired the ability to regulate other DNA repair, antiviral, and pathogen resistance genes.

### Do MER85s serve as a chromosomal reservoir for excess CSB-PGBD3 fusion protein?

Continued binding of the CSB-PGBD3 fusion protein to MER85s may be fortuitous but need not be functionless. SETMAR (also called Metnase) is another domesticated transposase that exhibits continued binding to dispersed copies of the parental transposon. SETMAR consists of a SET methyltransferase domain fused to a Mariner (Hsmar1) transposase domain. SETMAR has been shown to play a role in NHEJ (nonhomologous end joining) repair of double-stranded DNA breaks [55] as well as repairing and restarting damaged replication forks [56], but it also retains the ability to bind Mariner transposon TIR sequences [57]. The binding affinity of SETMAR for Mariner elements appears to be regulated by interactions with a damage-regulated partner protein, Pso4 [58]; although normally bound to Mariner elements, SETMAR is released in response to DNA damage [59]. Similarly, MER85s could serve as reservoirs for excess CSB-PGBD3, perhaps regulating the interactions of CSB-PGBD3 with AP-1 factors, or ensuring that CSB-PGBD3 is readily available throughout the genome (Figure 12).

### The role of the CSB-PGBD3 fusion protein in Cockayne syndrome

CS1AN cells are derived from a Cockayne syndrome patient with compound heterozygous CSB alleles. An early truncating mutation (K377term) in one CSB allele prevents expression of CSB and the CSB-PGBD3 fusion protein, but the 100 bp deletion in exon 13 of the other CSB allele [60] is located far downstream of PGBD3 and allows continued expression of the CSB-PGBD3 fusion protein in the absence of full-length CSB [10]. Surprisingly, genes downregulated by expression of full-length CSB in CS1AN cells [9] correlate strongly with CSB-PGBD3 binding sites in CSB-null UVSS1KO cells (Table S4). Thus, CSB-PGBD3 contributes to an aberrant transcriptional state in CS1AN cells by binding near, and perhaps interacting directly with, genes that are normally repressed by full-length CSB.

How could the N-terminal domain of CSB in the CSB-PGBD3 fusion protein activate genes that are normally repressed by CSB? One tantalizing but highly speculative scenario would be that for CSB-regulated genes, the autoinhibitory N-terminal domain of CSB [13] has dual checkpoint and transcriptional activation functions: Once the ATPase domain had engaged as a chromatin remodeler, the N-terminal domain would be released to activate transcription. Unconstrained in the fusion protein by the mutually autoinhibitory ATPase domain of CSB, the N-terminal CSB domain of CSB-PGBD3 would function as a constitutive transcriptional activator of CSB-regulated (and perhaps other) genes unless displaced by functional CSB.

GREAT analysis provided considerable insight into the consequences of the interactions of CSB-PGBD3 with TRE and TEAD1 motifs: Genes downregulated by CSB rescue of CS1AN cells correlate strikingly, for all regulatory domain sizes, with the entire set of CSB-PGBD3 binding sites including those with TRE, TEAD1, or no detectable motifs (green cells in Table S4). Thus, CSB-PGBD3 binding to each of these motifs, and even to the large number of peaks for which we could not identify a motif, correlates with upregulation of gene expression in CS1AN cells that continue to make the CSB-PGBD3 fusion protein but lack functional CSB. The correlation of CSB-PGBD3 binding sites with genes repressed by CSB in CS1AN cells suggests that the fusion protein substantially reshapes the transcriptome in CS patient CS1AN, and may do so in other CS patients whose mutations allow continued expression of the CSB-PGBD3 fusion protein in the absence of functional CSB.

Just as expression of functional CSB in CS1AN cells represses genes upregulated by continued expression of the CSB-PGBD3 fusion protein [9], so expression of the CSB-PGBD3 fusion protein in CSB-null UVSS1KO cells induces a strong interferon-related innate antiviral immune response which is dramatically repressed by coexpression of functional CSB [16]. This could be driven by CSB-PGBD3 binding to AP-1 binding motifs, which are known to play a role in upregulating pro-inflammatory cytokines [61] and chemokines such as IL-8 [62]. In normal aging, inflammation is driven by an increase in cytokine expression [63] and appears to be responsible for many age-related diseases [64]. Thus induction of AP-1 dependent inflammatory pathways by the CSB-PGD3 fusion protein may contribute to segmental aging in CS [65], and could be responsible for parts of the innate immune response (including IL-8) induced by CSB-PGBD3 expression in CSB-null UVSS1KO cells [16].

These observations suggest a previously unappreciated role for CSB in regulation of innate immunity and inflammation. Indeed, even CS patients who do not express the CSB-PGBD3 fusion protein because of mutations upstream of intron 5 (Figure 1) might inappropriately activate or fail to deactivate innate immune pathways. As perceptively advocated by Brooks et al. [66], inflammation and calcification of the brain are seen both in CS and in another childhood neurodevelopmental disease known as Aicardi-Goutiéres syndrome (AGS). In AGS, loss of RNASEH2 or TREX1 nuclease activity causes accumulation of intracellular DNA and RNA fragments, counterfeiting a viral infection and triggering a constitutive type I interferon response [67]. Our data suggest that CS may also have an autoimmune component, caused both by loss of downregulation through CSB, and inappropriate upregulation by the CSB-PGBD3 fusion protein. If so, CS patients may benefit from treatment with immunosuppressive or anti-inflammatory drugs.

## Materials and Methods

### Identification of MER85 locations

We previously identified the locations of 613 partial or complete MER85 elements [10]. Closer examination of these elements revealed that almost all of them are actually complete, with both 5′ and 3′ terminal inverted repeats (TIRs). Additional MER85 elements in the hg18 build of the human genome were obtained from the RepeatMasker 3.2.7 track [68] in the UCSC genome browser [45]. Several additional elements were located using the BLAT tool [69] in the UCSC genome browser with the 100 5′-most bases of the PGBD3 transposon as the query. Each MER85 element was examined individually to determine the boundaries of the sequence, the orientation, and the location of the TIRs and internal palindrome motifs (Table S1).

### Clones and cell lines

The HT1080 human fibrosarcoma cell line was maintained in Minimum Essential Medium Alpha (MEM-α) with 5% fetal bovine serum (FBS), penicillin, and streptomycin. All UVSS1KO-derived cell lines were cultured in Dulbecco's Modified Eagle Medium (DMEM) supplemented with 10% FBS, penicillin, and streptomycin. UVSS1KO cells stably expressing the pFLAG-HA-CSB, pFLAG-HA-CSB-PGBD3 and pFLAG-HA constructs have been described previously [16]. To generate analogous pFLAG-HA-CSB-eGFP, pFLAG-HA-CSB-LacI, and pFLAG-HA-eGFP-PGBD3 constructs, the indicated coding sequences were fused in frame and inserted into the same bicistronic pIREShyg3 backbone (Clontech); LacI was a gift of N. Maizels. The constructs were linearized before transfection into UVSS1KO cells (TransIT-LT1 transfection reagent, Mirus #MIR2300) and selection of stable pools with 200 µg/ml of hygromycin.

### Chromatin preparation

For ChIP-PCR, HT1080 cells were crosslinked with 1% formaldehyde for 10 minutes before quenching with 125 mM glycine. For ChIP-seq, UVSS1KO cells expressing FLAG-HA-CSB-PGBD3 were crosslinked with 0.5% formaldehyde for 5 min before quenching. Lower crosslinking was used for ChIP-seq to allow more thorough shearing of chromatin by sonication. Cells were then washed twice with phosphate buffered saline (PBS), scraped from the tissue culture plates, and resuspended in 1 ml cell lysis buffer (CLB, 5 mM PIPES pH 8, 85 mM KCl, 0.5% NP40) per 2×10^7^ cells. Cells in lysis buffer were vortexed for 10 sec, incubated on ice for 10 min, and vortexed again for 10 sec. After lysis, nuclei were pelleted by centrifugation and resuspended in 500 µl RIPA buffer (10 mM TrisHCl pH 8, 140 mM NaCl, 1% Triton X-100, 0.1% SDS, 0.1% deoxycholate, 0.1 mM EDTA, 0.05 mM EGTA) per 2×10^7^ cells. Glass beads (50 mg per 2×10^7^ cells) were added to assist shearing, and nuclei were broken by 6 pulses of 10 sec each from a Sonic Dismembrator (Fisher Scientific) at a setting of 4 W. Chromatin samples were precleared by nutation for 1 h at 4°C with crosslinked Staph A cells (20 µl per 2×10^7^ cells). Glass beads and Staph A were removed before immunoprecipitation.

### ChIP–PCR

Rabbit polyclonal antibodies specific for the N-terminal 240 residues and the C-terminal 158 residues of CSB were raised against fusions with bacterial GST [70]. CSB antibodies were purified from GST antibodies by passage over a GST column [10]. Rabbit anti-mouse IgG, rabbit polyclonal anti-CSB-N-terminus, rabbit polyclonal anti-CSB-C-terminus, or no antibody was added to HT1080 chromatin preparations at a dilution of 1∶200 and nutated overnight at 4°C. To precipitate bound antibodies, 0.1 vol Protein A-sepharose CL4B beads (Sigma) was added, and nutated for 1 h at 4°C. The beads were then washed 3x in RIPA buffer, 1x in RIPA wash buffer (10 mM Tris-HCl, pH 8, 250 mM LiCl, 0.1 mM EDTA, 0.5% NP40, 0.5% deoxycholate), and resuspended in 0.2 vol TE at pH 7.5. ChIP samples were digested with pancreatic ribonuclease A, followed by Proteinase K, and decrosslinked by incubation at 65°C overnight. ChIPs were assayed using PCR primers for the 6 genomic MER85 elements (Table S7) and α-^32^P-dCTP to body-label the products.

### ChIP–seq library preparation

ChIPs were performed as described for ChIP-PCR but using a 1∶200 dilution of mouse monoclonal 1B1 directed against the N-terminus of CSB (kind gift of Hua-Ying Fan, University of Pennsylvania) and Protein G Dynabeads (Invitrogen) for the pulldown. An input sample of sheared, crosslinked chromatin was set aside from the same chromatin pool used for ChIPs. The input sample was digested with RNase, protease, and decrosslinked without enrichment by ChIP. The ends of the ChIP and input samples were repaired using End-It (Epicentre), A-tailed using Taq polymerase (Invitrogen), and Illumina paired-end sequencing adapters were ligated using Quick T4 DNA Ligase (NEB). DNA fragments ranging from 400 to 700 bp were selected and purified by PAGE, then preamplified for 9 (input) or 12 cycles (ChIP) using Illumina paired-end preamplification primers, a BioRad iTaq supermix, and the following PCR protocol: denaturation 5 min at 95°C; cycling 30 sec at 95°C, 2 sec at 55°C, 2 sec at 72°C, and extension 10 sec at 72°C. For Illumina adapter and primer sequences, see supplementary methods of [27]. The preamplified samples were purified using a Qiagen PCR Cleanup kit, and were sequenced using an Illumina Genome Analyzer II (J. Shendure, University of Washington). Bases were called suing Illumina Real Time Analysis 1.5 software. Raw reads and processed data can be accessed at GEO study GSE37919.

### ChIP–seq read alignment

The input sample generated 4,735,921 reads of 36 bp each. Reads from a single end of each sequenced fragment were aligned to the UCSC build hg18 (NCBI36) using the read alignment program Bowtie v0.12.7 with settings -n 0, -m 1, and –best to ensure that mapped reads had no mismatches, did not match multiple locations in the genome, and were from the best stratum of alignments [28]. All together, 3,307,313 reads were successfully aligned. The CSB-PGBD3 ChIP sample generated 14,263,776 paired-end reads of 36 bp each. These reads were aligned using the same settings as for the input, but were aligned as pairs with default settings for read spacing. All together, 8,574,668 paired-end reads were successfully aligned. Alignment files were created in both Bowtie and SAM format for subsequent analysis. After using IGVTools to sort the SAM-formatted files, the paired-end SAM-formatted Bowtie output was converted using a Perl script into fragment overlap WIG files (available at http://code.google.com/p/graylt-plosgenetics-2012/) for use with the Cis-regulatory Element Annotation System (CEAS) and for subsequent analysis of fragment overlaps. The Perl script used paired-end reads as boundaries for each sequenced fragment, looked for clusters of at least 5 overlapping fragments of a specified size, and then calculated the number of sequenced fragments overlapping each position of the genome within each fragment cluster.

### ChIP–seq peak calling

ChIP-seq peaks were called using three peak-calling programs. Model-Based Analysis of ChIP-seq (MACS) was used to call peaks using the full paired-end CSB-PGBD3 ChIP and Input datasets [29]. MACS identified 45,067 peaks with a p-value<1e-5. For subsequent analysis, only the 9,835 peaks with a p-value<1e-12 were considered. ERANGE was also used for calling peaks after converting Bowtie map files to RDS format using the ERANGE makerdsfrombowtie Python script [30]. The ERANGE setting -minimum 2 was used to adjust the minimum enrichment threshold to 2-fold because of the disparate read depth between input and enriched samples. Using this setting, ERANGE found 3,743 peaks, which were used for subsequent analysis. The third peak-calling algorithm used was Quantitative Enrichment of Sequence Tags (QuEST), with settings for transcription factor ChIP and custom peak calling parameters (20, 10, 3) [31]. QuEST identified 5,663 peaks, which were used for subsequent analysis. Peaks from each of the three peak-calling algorithms were compared using the Join on Genomic Intervals feature of the multi-purpose Galaxy analysis tool [71]–[73]. Comparison of peaks from all three algorithms showed 2,087 distinct enriched regions (“combined peaks”) identified using all three algorithms. The 5′ and 3′ boundaries of these regions were determined based on the outermost boundaries of overlapping peaks identified using MACS, ERANGE, and QuEST. The summit of each combined peak was determined by using a Perl script to search for the center of the deepest fragment overlap in the CSB-PGBD3 WIG file generated as described above (available at http://code.google.com/p/graylt-plosgenetics-2012/). Combined peaks and peak summits are listed in (Table S2).

### Cumulative overlaps over CSB-PGBD3 binding elements

Galaxy's Intersect tool was used to compare the locations of 2,087 Combined Peaks to the locations of the 889 MER85 elements (Table S1). Peaks were found to overlap 363 MER85 elements. The cumulative fragment overlap over all 363 bound MER85 elements was calculated using a Perl script (available at http://code.google.com/p/graylt-plosgenetics-2012/) to sum the fragment overlaps over each MER85 element in the whole-genome CSB-PGBD3 fragment overlap WIG file after correcting the positions of the fragments for MER85 position and orientation. The same script was used to generate fragment overlap profiles over individual PGBD3 and PGBD3 pseudogenes, as well as sets of all bound TRE, TEAD1, and CTCF motifs.

### Identification of non-MER85 motifs

Combined peaks were filtered to remove peaks over MER85 elements using Galaxy's Subtract tool [71]. Using Galaxy's Extract Genomic DNA tool [71], 100 bp regions around each filtered peak summit (summit - 50 bp to summit +49 bp) were extracted [71] and then searched using a local installation of the Multiple-Em for Motif Elicitation (MEME) tool with the settings -dna -mod zoops -minw 6 -maxw 12 -revcomp -nmotifs 5 [74]. The Position Specific Frequency Matrix (PSFM) for each of the 5 statistically significant motifs identified by MEME were submitted to the online version of Tomtom [35] for comparison to motifs in the JASPAR and TRANSFAC transcription factor motif databases. Of the 5 motifs, 3 matched known transcription factors AP-1, TEAD1, and CTCF. Using Galaxy's Subtract tool, 892 peaks were identified that contained none of the 3 transcription factor motifs. The summit sequences of these peaks were resubmitted to MEME to identify any additional motifs that may have been masked by the high-scoring AP-1, TEAD1, or CTCF motifs, but no additional motifs were identified.

### Correlation of ChIP–seq peaks with UVSS1KO and CS1AN microarray datasets

Probe locations for all Affymetrix Human U133plus2.0 array probe sets were retrieved from the HG-U133_Plus_2 Annotations, CSV format, Release 31 (8/23/10) available on the Affymetrix web site. We had previously defined probe sets that were up- or downregulated at least 2-fold by expression of FLAG-HA-tagged CSB, CSB-PGBD3, or both compared to FLAG-HA tags alone in UVSS1KO cell lines [16], or by CSB rescue of the CS1AN cell line [9]. Lists of Affymetrix probes for each condition and the direction of expression change were compiled, and a Perl script used to convert each list of probes to a list of 5′ ends of probe set locations for regulated probe sets. These “probe set start sites” were converted to regulatory domains using the GREAT createRegulatoryDomains program locally with settings for 1 Mb, 250 kb, or 100 kb maximum extensions [41]. The regulatory domains were then compared to sets of peaks using a Perl script that counted overlaps between the peak summits and the set of regulatory domains (available at http://code.google.com/p/graylt-plosgenetics-2012/). These counts were submitted to the GREAT calculateBinomialP program locally to obtain P-values (Table S4). False discovery rates (FDR) were then calculated empirically for each comparison using 100 sets of randomly selected summit locations of the same size as each peak set. P-values corresponding to a FDR below 1% were considered significant. Finally, the peak summits were compared to the regulatory domain tables that include gene names, and lists of genes with nearby CSB-PGBD3 peaks were generated for each comparison.

### Comparison of CSB-PGBD3 peaks to diverse gene ontologies

Peak summit locations for all identified peaks, as well as for MER85, AP-1, TEAD1, CTCF, and other peaks separately, were submitted to GREAT v1.8.2 (great.stanford.edu) using default association rule settings for the hg18 genome build. The results from each analysis, including up to 20 significantly enriched gene sets for each database with a region enrichment of 2-fold or more and an FDR<0.05, were downloaded as tab-separated files.

### Detection of AP-1 protein expression

Whole cell lysates from UVSS1KO cell lines were separated by SDS-PAGE and western blotted as described previously [10]. Primary antibodies were rabbit polyclonal anti-JunD (sc-74, Santa Cruz Biotechnology), rabbit polyclonal anti-c-Jun (sc-1694, Santa Cruz Biotechnology), mouse monoclonal anti-Fra-1 (sc-28310, Santa Cruz Biotechnology), rabbit polyclonal anti-Fra-2 (sc-13017, Santa Cruz Biotechnology), mouse monoclonal ANTI-FLAG M2 (F3165, Sigma-Aldrich), and mouse monoclonal anti-actin (A2228, Sigma-Aldrich).

### Co-immunoprecipitations

Subconfluent cells were trypsinized, washed in PBS, and counted. Nuclei were prepared by detergent lysis in CLB (1 ml/10^7^ cells) and pelleted after 10 min on ice. Whole cells or nuclei were resuspended in IP50 buffer [75] (10 mM TrisHCl, 50 mM KCl, 0.1 mM EDTA, 10% glycerol, 1× protease inhibitor cocktail [Roche]) at a concentration of 10^6^ nuclei/ml and sonicated for 10 sec using a Sonic Dismembrator (Fischer Scientific) at 4 W. Aliquots of 0.5 ml containing 5×10^6^ cells or nuclei were nutated with a 1∶200 dilution of antibody for 1 h, followed by a pulldown with Protein G Dynabeads (Invitrogen). The beads were washed 3 times with IP50 buffer, then resuspended in sample buffer, denatured, and resolved by 6% SDS PAGE (for RNAPII and FLAG-HA tagged proteins) or 10% SDS PAGE (for c-Jun). The gels were electroblotted to PVDF membranes, and blocked with 5% nonfat dry milk in Tris-buffered saline (TBS). Primary antibodies were added in blocking buffer containing 0.1% Tween 20, and the membrane washed 3× with TBS containing 0.1% Tween 20 (TBST). Horseradish Peroxidase (HRP)-coupled secondary antibodies were added in blocking buffer with Tween, then washed 3× with TBST. The HRP signal was detected on X-ray film using ECL Plus Western Blot Detection reagents (GE Healthcare).

### Quantitative PCR

Total RNA was extracted from UVSS1KO-derived cell lines using TRIzol (Invitrogen). RNAs were reverse-transcribed using random primers (Invitrogen) and Superscript III (Invitrogen), then digested with pancreatic RNase A. cDNAs were purified on PCR Cleanup columns (Qiagen), quantified with a Nanodrop spectrophotometer, and used for QPCR. QPCR was performed using SYBR Green Master Mix (BioRad) with 1.25 µM primers and 20 ng cDNA per reaction. All 4 combinations of 2 forward and 2 reverse primers were tested in the QPCR protocol. Table S8 lists primer pairs that amplified under QPCR conditions, generated a clean melting curve, and produced a single band upon gel electrophoresis of QPCR products.

### Electrophoretic mobility shift assays

PGBD3 was expressed and MER85s were cloned as described in [16]. Electrophoretic mobility shift assays (EMSAs) were performed as described in [76]. Gels were dried, used to expose a storage phosphor screen, and scanned using a phosphorimager. Images were then quantitated using ImageJ [77], and the difference in intensity of shifted bands was compared between adjascent lanes from samples with and without addition of PGBD3 protein. These differences were then normalized by comparison with the scrambled sequence control (0%) and the MER85 consensus control (100%).

### Identification of transcription factor binding sites in MER85 elements

Locations of MER85-39, MER85-236, MER85-592, and MER85-763 were converted to hg19 coordinates using the UCSC Genome Browser Convert tool, then were submitted to the online version of MAPPER2 [78] to locate transcription factor binding sites from the JASPAR database. Results were filtered to the 90^th^ percentile of MAPPER scores, then displayed in the UCSC Genome Browser using a feature of the MAPPER2 website. Transcription factor binding sites found in 3 of 4 MER85s were then used to search the Entrez Gene database [79] for human homologues.

## Supporting Information

Figure S1 The PGBD3 transposase is not capable of binding directly to TRE motifs *in vitro*. For EMSA assays, purified PGBD3 transposase was mixed with end-labeled 42 bp duplex oligonucleotides containing Repbase consensus MER85 sequence, 1 or 2 tumor promoting antigen response element (TRE) motifs, or random sequence.
(TIF)

Figure S2 Fragment overlaps over all full-length PGBD3 insertions in the genome, including all four PGBD3 pseudogenes, correlate with conserved palindrome sequences. (a) Fragment overlap binding profiles over PGBD3 and each of the PGBD3 pseudogenes. (b) Sequences of the TIR and palindromes of each of PGBD3 and each of the pseudogenes. Mismatches with respect to the PGBD3 p1 sequence are in bold. TIR, terminal inverted repeat; NP, sequence not present in truncated PGBD3 pseudogene.
(TIF)

Figure S3 CSB-PGBD3 fusion protein is enriched near gene promoters, but most peaks are distal and intronic. The Cis-regulatory Element Annotation (CEAS) Tool was used to generate a gene-centered annotation of 2,087 CSB-PGBD3 peaks found in common by MACS, ERANGE, and QuEST. Promoter regions include 3 kb upstream of the transcription start site (TSS). Downstream regions include 3 kb beyond the polyadenylation site. P-values generated by CEAS for overrepresentation of CSB-PGBD3 binding are shown in parentheses.
(TIF)

Figure S4 c-Jun, JunD, and Fra2 are expressed in UVSS1KO cell lines. Lysates from UVSS1KO cells expressing FLAG-HA tags or FLAG-HA-tagged CSB-PGBD3, CSB, CSB-eGFP, or eGFP-PGBD3 were western blotted for expression of JunD, c-Jun, Fra2, actin and FLAG-HA-tagged proteins.
(TIF)

Figure S5 MER85 elements contain potential transcription factor binding sites. Locations of transcription factor binding sites from the JASPAR database found in 4 MER85 elements using MAPPER2 and displayed in the UCSC Genome Browser. MER85 sequences are shown from the upstream to downstream target site duplication (TSD).
(TIF)

Table S1 Locations and characteristics of PGBD3, 4 PGBD3 pseudogenes, and 889 MER85 elements in the hg18 genome. Sequences of the PGBD3 and MER85 elements, the 5′ and 3′ TIRs, and the palindrome are listed along with the peak ID from Table S2 for PGBD3s and MER85s that bound the CSB-PGBD3 fusion protein.
(XLS)

Table S2 The CSB-PGBD3 fusion protein is enriched at >2,000 locations in the hg18 human genome build. CSB-PGBD3 peaks were identified by all three peak finders (ERANGE, MACS, and QuEST) as significantly enriched by immunoprecipitation compared to the Input control. Summit locations were calculated as the region of greatest fragment overlap within the peak.
(XLS)

Table S3 Genomic locations of TRE, TEAD1, and CTCF motifs identified by MEME within 50 bp of CSB-PGBD3 peak summits in the hg18 genome.
(XLS)

Table S4 Summary of GREAT comparisons between CSB-PGBD3 binding sites and genes that exhibit expression changes of 2-fold or more when CSB, CSB-PGBD3, or both were stably expressed in CSB-null UVSS1KO cells, or when wild type CSB was stably expressed in the compound heterozygous CS1AN cell line which continues to express the CSB-PGBD3 fusion protein. The number of peaks in each set are given by numbers in parentheses. Bold p-values indicate a false discovery rate of less than 1%.
(XLS)

Table S5 GREAT results for comparisons of CSB-PGBD3 binding sites to diverse sets of gene ontologies. Only the five most significant results displayed by GREAT for each category are presented. Angiogenesis and blood vessel development genes are highly enriched near binding sites containing TRE or TEAD1 motifs. AP-1 proteins are known to regulate genes related to angiogenesis [18] although a role for TEAD1 in this process has not been studied. Genes involved in the related, but distinct transforming growth factor beta (TGF-beta) and bone morphogenesis protein (BMP) receptor signaling pathways are enriched near CSB-PGBD3 binding sites. The BMP receptor pathway is significantly enriched near the set of all CSB-PGBD3 peaks and the set with no identified motif, whereas TGF-beta receptor signaling is enriched near bound TRE motifs. SMAD2/3 signaling and the ALK1 pathway are also enriched near CSB-PGBD3 peaks over TRE motifs. All four of these factors – BMPR, TGF-beta, SMAD2/3, and ALK1 – are involved in overlapping pathways that regulate cell proliferation, bone growth, angiogenesis, and cell migration [80], [81]. Disease Ontology and MSigDB perturbation terms related to breast, osteosarcoma, ovarian, cervical, melanocytic, and pancreatic cancers as well as GO Biological Processes for extracellular matrix organization and regulation of cell adhesion are enriched in one or multiple peak categories consistent with the notion that these ontologies reflect common pathways in oncogenesis. Immune response genes are enriched near CSB-PGBD3 binding sites, particularly those involved in IL-2 and IFN-gamma signaling. CSB-PGBD3 binding sites with TRE and TEAD1 motifs correlate strongly with IFN-gamma ontologies from Pathway Commons, while the set of all CSB-PGBD3 peaks and peaks with no identified motif were enriched near genes related to IL-2 signaling. GO, Gene Ontology; MSigDB, Molecular Signatures Database. *Raw binomial P-values reported by GREAT. **Hypergeometric false discovery rate (FDR) reported by GREAT.
(XLS)

Table S6 105 CSB-PGBD3 peaks overlap at least 10 of 18 RNAPII peak datasets from 15 cell lines available in the UCSC Genome Browser database. The number of RNAPII datasets for which each CSB-PGBD3 peak overlaps an RNAPII peak are tabulated, and individual overlaps are annotated as 1 where an overlap was detected and 0 where no overlap was found. Peak IDs correspond to peak locations in Table S2.
(XLS)

Table S7 Primers used for ChIP-PCR of genomic MER85 elements.
(XLS)

Table S8 Primers used for QPCR of selected cDNA targets.
(XLS)

## References

[1] Laugel V, Dalloz C, Durand M, Sauvanaud F, Kristensen U, et al (2010) Mutation update for the CSB/ERCC6 and CSA/ERCC8 genes involved in Cockayne syndrome. Hum Mutat 31: 113–126 doi:10.1002/humu.21154. 19894250 10.1002/humu.21154

[2] Tantin D, Kansal A, Carey M (1997) Recruitment of the putative transcription-repair coupling factor CSB/ERCC6 to RNA polymerase II elongation complexes. Mol Cell Biol 17: 6803–6814. 9372911 10.1128/mcb.17.12.6803 PMC232536

[3] van den Boom V, Citterio E, Hoogstraten D, Zotter A, Egly J-M, et al (2004) DNA damage stabilizes interaction of CSB with the transcription elongation machinery. J Cell Biol 166: 27–36 doi:10.1083/jcb.200401056. 15226310 10.1083/jcb.200401056 PMC2172148

[4] van Gool AJ, Citterio E, Rademakers S, van Os R, Vermeulen W, et al (1997) The Cockayne syndrome B protein, involved in transcription-coupled DNA repair, resides in an RNA polymerase II-containing complex. EMBO J 16: 5955–5965 doi:10.1093/emboj/16.19.5955. 9312053 10.1093/emboj/16.19.5955 PMC1170226

[5] Lagerwerf S, Vrouwe MG, Overmeer RM, Fousteri MI, Mullenders LHF (2011) DNA damage response and transcription. DNA Repair (Amst) 10: 743–750 doi:10.1016/j.dnarep.2011.04.024. 21622031 10.1016/j.dnarep.2011.04.024

[6] Anindya R, Mari P-O, Kristensen U, Kool H, Giglia-Mari G, et al (2010) A ubiquitin-binding domain in Cockayne syndrome B required for transcription-coupled nucleotide excision repair. Mol Cell 38: 637–648 doi:10.1016/j.molcel.2010.04.017. 20541997 10.1016/j.molcel.2010.04.017 PMC2885502

[7] Gray LT, Weiner AM (2010) Ubiquitin recognition by the Cockayne syndrome group B protein: binding will set you free. Mol Cell 38: 621–622 doi:10.1016/j.molcel.2010.05.025. 20541993 10.1016/j.molcel.2010.05.025

[8] Yuan X, Feng W, Imhof A, Grummt I, Zhou Y (2007) Activation of RNA polymerase I transcription by cockayne syndrome group B protein and histone methyltransferase G9a. Mol Cell 27: 585–595 doi:10.1016/j.molcel.2007.06.021. 17707230 10.1016/j.molcel.2007.06.021

[9] Newman JC, Bailey AD, Weiner AM (2006) Cockayne syndrome group B protein (CSB) plays a general role in chromatin maintenance and remodeling. Proc Natl Acad Sci USA 103: 9613–9618 doi:10.1073/pnas.0510909103. 16772382 10.1073/pnas.0510909103 PMC1480455

[10] Newman JC, Bailey AD, Fan H-Y, Pavelitz T, Weiner AM (2008) An abundant evolutionarily conserved CSB-PiggyBac fusion protein expressed in Cockayne syndrome. PLoS Genet 4: e1000031 doi:10.1371/journal.pgen.1000031. 18369450 10.1371/journal.pgen.1000031 PMC2268245

[11] Brosh RM, Balajee AS, Selzer RR, Sunesen M, Proietti De Santis L, et al (1999) The ATPase domain but not the acidic region of Cockayne syndrome group B gene product is essential for DNA repair. Mol Biol Cell 10: 3583–3594. 10564257 10.1091/mbc.10.11.3583 PMC25641

[12] Sunesen M, Selzer RR, Brosh RM, Balajee AS, Stevnsner T, et al (2000) Molecular characterization of an acidic region deletion mutant of Cockayne syndrome group B protein. Nucleic Acids Research 28: 3151–3159. 10931931 10.1093/nar/28.16.3151 PMC108419

[13] Lake RJ, Geyko A, Hemashettar G, Zhao Y, Fan H-Y (2010) UV-induced association of the CSB remodeling protein with chromatin requires ATP-dependent relief of N-terminal autorepression. Mol Cell 37: 235–246 doi:10.1016/j.molcel.2009.10.027. 20122405 10.1016/j.molcel.2009.10.027 PMC2818792

[14] Lebedev A, Scharffetter-Kochanek K, Iben S (2008) Truncated Cockayne syndrome B protein represses elongation by RNA polymerase I. J Mol Biol 382: 266–274 doi:10.1016/j.jmb.2008.07.018. 18656484 10.1016/j.jmb.2008.07.018

[15] Horibata K, Saijo M, Bay MN, Lan L, Kuraoka I, et al (2011) Mutant Cockayne syndrome group B protein inhibits repair of DNA topoisomerase I-DNA covalent complex. Genes Cells 16: 101–114 doi:10.1111/j.1365-2443.2010.01467.x. 21143350 10.1111/j.1365-2443.2010.01467.x

[16] Bailey AD, Gray LT, Pavelitz T, Newman JC, Horibata K, et al (2012) The conserved Cockayne syndrome B-piggyBac fusion protein (CSB-PGBD3) affects DNA repair and induces both interferon-like and innate antiviral responses in CSB-null cells. DNA Repair (Amst) doi:10.1016/j.dnarep.2012.02.004. 10.1016/j.dnarep.2012.02.004 PMC3340519 22483866

[17] Dong W, Li Y, Gao M, Hu M, Li X, et al (2011) IKK contributes to UVB-induced VEGF expression by regulating AP-1 transactivation. Nucleic Acids Research doi:10.1093/nar/gkr1216. 10.1093/nar/gkr1216 PMC3326327 22169952

[18] Shan Z-X, Lin Q-X, Yang M, Zhang Bin, Zhu J-N, et al (2011) Transcription factor Ap-1 mediates proangiogenic MIF expression in human endothelial cells exposed to Angiotensin II. Cytokine 53: 35–41 doi:10.1016/j.cyto.2010.09.009. 21030269 10.1016/j.cyto.2010.09.009

[19] Zenz R, Eferl R, Scheinecker C, Redlich K, Smolen J, et al (2008) Activator protein 1 (Fos/Jun) functions in inflammatory bone and skin disease. Arthritis Res Ther 10: 201 doi:10.1186/ar2338. 18226189 10.1186/ar2338 PMC2374460

[20] Zhang Y, Feng XH, Derynck R (1998) Smad3 and Smad4 cooperate with c-Jun/c-Fos to mediate TGF-beta-induced transcription. Nature 394: 909–913 doi:10.1038/29814. 9732876 10.1038/29814

[21] Jurka J, Kapitonov VV, Pavlicek A, Klonowski P, Kohany O, et al (2005) Repbase Update, a database of eukaryotic repetitive elements. Cytogenet Genome Res 110: 462–467 doi:10.1159/000084979. 16093699 10.1159/000084979

[22] Cary LC, Goebel M, Corsaro BG, Wang HG, Rosen E, et al (1989) Transposon mutagenesis of baculoviruses: analysis of Trichoplusia ni transposon IFP2 insertions within the FP-locus of nuclear polyhedrosis viruses. Virology 172: 156–169. 2549707 10.1016/0042-6822(89)90117-7

[23] Li X, Lobo N, Bauser CA, Fraser MJ (2001) The minimum internal and external sequence requirements for transposition of the eukaryotic transformation vector piggyBac. Mol Genet Genomics 266: 190–198. 11683259 10.1007/s004380100525

[24] Yusa K, Zhou L, Li MA, Bradley A, Craig NL (2011) A hyperactive piggyBac transposase for mammalian applications. Proc Natl Acad Sci USA 108: 1531–1536 doi:10.1073/pnas.1008322108. 21205896 10.1073/pnas.1008322108 PMC3029773

[25] Whitfield CR, Shilton BH, Haniford DB (2012) Identification of basepairs within Tn5 termini that are critical for H-NS binding to the transpososome and regulation of Tn5 transposition. Mob DNA 3: 7 doi:10.1186/1759-8753-3-7. 22503096 10.1186/1759-8753-3-7 PMC3347997

[26] Horibata K, Iwamoto Y, Kuraoka I, Jaspers NGJ, Kurimasa A, et al (2004) Complete absence of Cockayne syndrome group B gene product gives rise to UV-sensitive syndrome but not Cockayne syndrome. Proc Natl Acad Sci USA 101: 15410–15415 doi:10.1073/pnas.0404587101. 15486090 10.1073/pnas.0404587101 PMC524447

[27] Bentley DR, Balasubramanian S, Swerdlow HP, Smith GP, Milton J, et al (2008) Accurate whole human genome sequencing using reversible terminator chemistry. Nature 456: 53–59 doi:10.1038/nature07517. 18987734 10.1038/nature07517 PMC2581791

[28] Langmead B, Trapnell C, Pop M, Salzberg SL (2009) Ultrafast and memory-efficient alignment of short DNA sequences to the human genome. Genome Biol 10: R25 doi:10.1186/gb-2009-10-3-r25. 19261174 10.1186/gb-2009-10-3-r25 PMC2690996

[29] Zhang Y, Liu T, Meyer CA, Eeckhoute J, Johnson DS, et al (2008) Model-based analysis of ChIP-Seq (MACS). Genome Biol 9: R137 doi:10.1186/gb-2008-9-9-r137. 18798982 10.1186/gb-2008-9-9-r137 PMC2592715

[30] Johnson DS, Mortazavi A, Myers RM, Wold B (2007) Genome-Wide Mapping of in Vivo Protein-DNA Interactions. Science 316: 1497–1502 doi:10.1126/science.1141319. 17540862 10.1126/science.1141319

[31] Valouev A, Johnson DS, Sundquist A, Medina C, Anton E, et al (2008) Genome-wide analysis of transcription factor binding sites based on ChIP-Seq data. Nat Methods 5: 829–834 doi:10.1038/nmeth.1246. 19160518 10.1038/nmeth.1246 PMC2917543

[32] Pepke S, Wold B, Mortazavi A (2009) Computation for ChIP-seq and RNA-seq studies. Nat Methods 6: S22–S32 doi:10.1038/nmeth.1371. 19844228 10.1038/nmeth.1371 PMC4121056

[33] Shin H, Liu T, Manrai AK, Liu XS (2009) CEAS: cis-regulatory element annotation system. Bioinformatics 25: 2605–2606 doi:10.1093/bioinformatics/btp479. 19689956 10.1093/bioinformatics/btp479

[34] Bailey TL, Elkan C (1994) Fitting a mixture model by expectation maximization to discover motifs in biopolymers. Proc Int Conf Intell Syst Mol Biol 2: 28–36. 7584402

[35] Gupta S, Stamatoyannopoulos JA, Bailey TL, Noble WS (2007) Quantifying similarity between motifs. Genome Biol 8: R24 doi:10.1186/gb-2007-8-2-r24. 17324271 10.1186/gb-2007-8-2-r24 PMC1852410

[36] Eferl R, Wagner EF (2003) AP-1: a double-edged sword in tumorigenesis. Nat Rev Cancer 3: 859–868 doi:10.1038/nrc1209. 14668816 10.1038/nrc1209

[37] Phillips JE, Corces VG (2009) CTCF: master weaver of the genome. Cell 137: 1194–1211 doi:10.1016/j.cell.2009.06.001. 19563753 10.1016/j.cell.2009.06.001 PMC3040116

[38] Wagner EF, Eferl R (2005) Fos/AP-1 proteins in bone and the immune system. Immunol Rev 208: 126–140 doi:10.1111/j.0105-2896.2005.00332.x. 16313345 10.1111/j.0105-2896.2005.00332.x

[39] Chinenov Y, Kerppola TK (2001) Close encounters of many kinds: Fos-Jun interactions that mediate transcription regulatory specificity. Oncogene 20: 2438–2452 doi:10.1038/sj.onc.1204385. 11402339 10.1038/sj.onc.1204385

[40] Lallemand D, Spyrou G, Yaniv M, Pfarr CM (1997) Variations in Jun and Fos protein expression and AP-1 activity in cycling, resting and stimulated fibroblasts. Oncogene 14: 819–830 doi:10.1038/sj.onc.1200901. 9047389 10.1038/sj.onc.1200901

[41] McLean CY, Bristor D, Hiller M, Clarke SL, Schaar BT, et al (2010) GREAT improves functional interpretation of cis-regulatory regions. Nat Biotechnol 28: 495–501 doi:10.1038/nbt.1630. 20436461 10.1038/nbt.1630 PMC4840234

[42] Storey J (2002) A direct approach to false discovery rates - Storey - 2002 - Journal of the Royal Statistical Society: Series B (Statistical Methodology) - Wiley Online Library. Journal of the Royal Statistical Society Series B …

[43] Pace JK, Feschotte C (2007) The evolutionary history of human DNA transposons: evidence for intense activity in the primate lineage. Genome Res 17: 422–432 doi:10.1101/gr.5826307. 17339369 10.1101/gr.5826307 PMC1832089

[44] Mitra R, Fain-Thornton J, Craig NL (2008) piggyBac can bypass DNA synthesis during cut and paste transposition. EMBO J 27: 1097–1109 doi:10.1038/emboj.2008.41. 18354502 10.1038/emboj.2008.41 PMC2323262

[45] Dreszer TR, Karolchik D, Zweig AS, Hinrichs AS, Raney BJ, et al (2012) The UCSC Genome Browser database: extensions and updates 2011. Nucleic Acids Research 40: D918–D923 doi:10.1093/nar/gkr1055. 22086951 10.1093/nar/gkr1055 PMC3245018

[46] Karolchik D (2004) The UCSC Table Browser data retrieval tool. Nucleic Acids Research 32: 493D–496 doi:10.1093/nar/gkh103. 10.1093/nar/gkh103 PMC308837 14681465

[47] Fan H-Y, Trotter KW, Archer TK, Kingston RE (2005) Swapping function of two chromatin remodeling complexes. Mol Cell 17: 805–815 doi:10.1016/j.molcel.2005.02.024. 15780937 10.1016/j.molcel.2005.02.024

[48] Ouyang X, Li J, Li G, Li B, Chen B, et al (2011) Genome-wide binding site analysis of FAR-RED ELONGATED HYPOCOTYL3 reveals its novel function in Arabidopsis development. Plant Cell 23: 2514–2535 doi:10.1105/tpc.111.085126. 21803941 10.1105/tpc.111.085126 PMC3226222

[49] Lynch VJ, Leclerc RD, May G, Wagner GP (2011) Transposon-mediated rewiring of gene regulatory networks contributed to the evolution of pregnancy in mammals. Nat Genet 43: 1154–1159 doi:10.1038/ng.917. 21946353 10.1038/ng.917

[50] Schmidt D, Schwalie PC, Wilson MD, Ballester B, Gonçalves A, et al (2012) Waves of retrotransposon expansion remodel genome organization and CTCF binding in multiple mammalian lineages. Cell 148: 335–348 doi:10.1016/j.cell.2011.11.058. 22244452 10.1016/j.cell.2011.11.058 PMC3368268

[51] Lake RJ, Basheer A, Fan H-Y (2011) Reciprocally regulated chromatin association of the Cockayne syndrome protein B and p53. J Biol Chem doi:10.1074/jbc.M111.252643. 10.1074/jbc.M111.252643 PMC3186407 21852235

[52] Dérijard B, Hibi M, Wu IH, Barrett T, Su B, et al (1994) JNK1: a protein kinase stimulated by UV light and Ha-Ras that binds and phosphorylates the c-Jun activation domain. Cell 76: 1025–1037. 8137421 10.1016/0092-8674(94)90380-8

[53] Reno EM, Haughian JM, Jackson TA, Thorne AM, Bradford AP (2009) c-Jun N-terminal kinase regulates apoptosis in endometrial cancer cells. Apoptosis 14: 809–820 doi:10.1007/s10495-009-0354-6. 19424800 10.1007/s10495-009-0354-6

[54] Ip JY, Schmidt D, Pan Q, Ramani AK, Fraser AG, et al (2011) Global impact of RNA polymerase II elongation inhibition on alternative splicing regulation. Genome Res 21: 390–401 doi:10.1101/gr.111070.110. 21163941 10.1101/gr.111070.110 PMC3044853

[55] Hromas R, Wray J, Lee S-H, Martinez L, Farrington J, et al (2008) The human set and transposase domain protein Metnase interacts with DNA Ligase IV and enhances the efficiency and accuracy of non-homologous end-joining. DNA Repair (Amst) 7: 1927–1937 doi:10.1016/j.dnarep.2008.08.002. 18773976 10.1016/j.dnarep.2008.08.002 PMC2644637

[56] De Haro LP, Wray J, Williamson EA, Durant ST, Corwin L, et al (2010) Metnase promotes restart and repair of stalled and collapsed replication forks. Nucleic Acids Research 38: 5681–5691 doi:10.1093/nar/gkq339. 20457750 10.1093/nar/gkq339 PMC2943610

[57] Liu D, Bischerour J, Siddique A, Buisine N, Bigot Y, et al (2007) The human SETMAR protein preserves most of the activities of the ancestral Hsmar1 transposase. Mol Cell Biol 27: 1125–1132 doi:10.1128/MCB.01899-06. 17130240 10.1128/MCB.01899-06 PMC1800679

[58] Beck BD, Lee SS, Hromas R, Lee S-H (2010) Regulation of Metnase's TIR binding activity by its binding partner, Pso4. Arch Biochem Biophys 498: 89–94 doi:10.1016/j.abb.2010.04.011. 20416268 10.1016/j.abb.2010.04.011 PMC2880194

[59] Beck BD, Park S-J, Lee Y-J, Roman Y, Hromas RA, et al (2008) Human Pso4 is a metnase (SETMAR)-binding partner that regulates metnase function in DNA repair. J Biol Chem 283: 9023–9030 doi:10.1074/jbc.M800150200. 18263876 10.1074/jbc.M800150200 PMC2431028

[60] Troelstra C, van Gool A, de Wit J, Vermeulen W, Bootsma D, et al (1992) ERCC6, a member of a subfamily of putative helicases, is involved in Cockayne's syndrome and preferential repair of active genes. Cell 71: 939–953. 1339317 10.1016/0092-8674(92)90390-x

[61] Bahar B, O'Doherty JV, Maher S, McMorrow J, Sweeney T (2012) Chitooligosaccharide elicits acute inflammatory cytokine response through AP-1 pathway in human intestinal epithelial-like (Caco-2) cells. Molecular Immunology 1–9 doi:10.1016/j.molimm.2012.03.027. 10.1016/j.molimm.2012.03.027 22512945

[62] Hipp MS, Urbich C, Mayer P, Wischhusen J, Weller M, et al (2002) AID-IMMU2208>3.0.CO;2-2.

[63] Orjalo AV, Bhaumik D, Gengler BK, Scott GK, Campisi J (2009) Cell surface-bound IL-1alpha is an upstream regulator of the senescence-associated IL-6/IL-8 cytokine network. Proc Natl Acad Sci USA 106: 17031–17036 doi:10.1073/pnas.0905299106. 19805069 10.1073/pnas.0905299106 PMC2761322

[64] Franceschi C, Bonafè M, Valensin S, Olivieri F, De Luca M, et al (2000) Inflamm-aging. An evolutionary perspective on immunosenescence. Ann N Y Acad Sci 908: 244–254. 10911963 10.1111/j.1749-6632.2000.tb06651.x

[65] Weidenheim KM, Dickson DW, Rapin I (2009) Neuropathology of Cockayne syndrome: Evidence for impaired development, premature aging, and neurodegeneration. Mech Ageing Dev 130: 619–636 doi:10.1016/j.mad.2009.07.006. 19647012 10.1016/j.mad.2009.07.006

[66] Brooks PJ, Cheng T-F, Cooper L (2008) Do all of the neurologic diseases in patients with DNA repair gene mutations result from the accumulation of DNA damage? DNA Repair (Amst) 7: 834–848 doi:10.1016/j.dnarep.2008.01.017. 18339586 10.1016/j.dnarep.2008.01.017 PMC2408373

[67] Gall A, Treuting P, Elkon KB, Loo Y-M, Gale M, et al (2012) Autoimmunity Initiates in Nonhematopoietic Cells and Progresses via Lymphocytes in an Interferon-Dependent Autoimmune Disease. Immunity 36: 120–131 doi:10.1016/j.immuni.2011.11.018. 22284419 10.1016/j.immuni.2011.11.018 PMC3269499

[68] Smit AFA, Hubley R, Green P (1996–2012) RepeatMasker Open-3.0. Available:http://www.repeatmasker.org.

[69] Kent WJ (2002) BLAT–the BLAST-like alignment tool. Genome Res 12: 656–664 doi:10.1101/gr.229202. 11932250 10.1101/gr.229202 PMC187518

[70] Yu A, Fan HY, Liao D, Bailey AD, Weiner AM (2000) Activation of p53 or loss of the Cockayne syndrome group B repair protein causes metaphase fragility of human U1, U2, and 5S genes. Mol Cell 5: 801–810. 10882116 10.1016/s1097-2765(00)80320-2

[71] Goecks J, Nekrutenko A, Taylor J (2010) Galaxy Team (2010) Galaxy: a comprehensive approach for supporting accessible, reproducible, and transparent computational research in the life sciences. Genome Biol 11: R86 doi:10.1186/gb-2010-11-8-r86. 20738864 10.1186/gb-2010-11-8-r86 PMC2945788

[72] Giardine B, Riemer C, Hardison RC, Burhans R, Elnitski L, et al (2005) Galaxy: a platform for interactive large-scale genome analysis. Genome Res 15: 1451–1455 doi:10.1101/gr.4086505. 16169926 10.1101/gr.4086505 PMC1240089

[73] Blankenberg D, Kuster Von G, Coraor N, Ananda G, Lazarus R, et al (2010) Galaxy: a web-based genome analysis tool for experimentalists. Curr Protoc Mol Biol Chapter 19: Unit19.10.1–Unit19.10.21 doi:10.1002/0471142727.mb1910s89. 10.1002/0471142727.mb1910s89 PMC4264107 20069535

[74] Bailey TL, Williams N, Misleh C, Li WW (2006) MEME: discovering and analyzing DNA and protein sequence motifs. Nucleic Acids Research 34: W369–W373 doi:10.1093/nar/gkl198. 16845028 10.1093/nar/gkl198 PMC1538909

[75] Bradsher J, Auriol J, Proietti-De-Santis L, Iben S, Vonesch JL, et al (2002) CSB is a component of RNA pol I transcription. Mol Cell 10: 819–829. 12419226 10.1016/s1097-2765(02)00678-0

[76] Ares M, Chung JS, Giglio L, Weiner AM (1987) Distinct factors with Sp1 and NF-A specificities bind to adjacent functional elements of the human U2 snRNA gene enhancer. Genes Dev 1: 808–817. 3428599 10.1101/gad.1.8.808

[77] Abramoff MD, Magalhaes PJ, Ram SJ (2004) Image Processing with ImageJ. Biophotonics International 11: 36–42.

[78] Marinescu VD, Kohane IS, Riva A (2005) MAPPER: a search engine for the computational identification of putative transcription factor binding sites in multiple genomes. BMC Bioinformatics 6: 79 doi:10.1186/1471-2105-6-79. 15799782 10.1186/1471-2105-6-79 PMC1131891

[79] Maglott D (2004) Entrez Gene: gene-centered information at NCBI. Nucleic Acids Research 33: D54–D58 doi:10.1093/nar/gki031. 10.1093/nar/gki031 PMC539985 15608257

[80] Kanehisa M, Goto S, Sato Y, Furumichi M, Tanabe M (2011) KEGG for integration and interpretation of large-scale molecular data sets. Nucleic Acids Research 40: D109–D114 doi:10.1093/nar/gkr988. 22080510 10.1093/nar/gkr988 PMC3245020

[81] Kanehisa M, Goto S (2000) KEGG: kyoto encyclopedia of genes and genomes. Nucleic Acids Research 28: 27–30. 10592173 10.1093/nar/28.1.27 PMC102409

