# Supplementary figures and images for: Tethering of the Conserved piggyBac Transposase Fusion Protein CSB-PGBD3 to Chromosomal AP-1 Proteins Regulates Expression of Nearby Genes in Humans
Source: PLoS Genet. 2012 Sep 27;8(9):e1002972. doi: 10.1371/journal.pgen.1002972 (PMC3459987; doi:10.1371/journal.pgen.1002972)

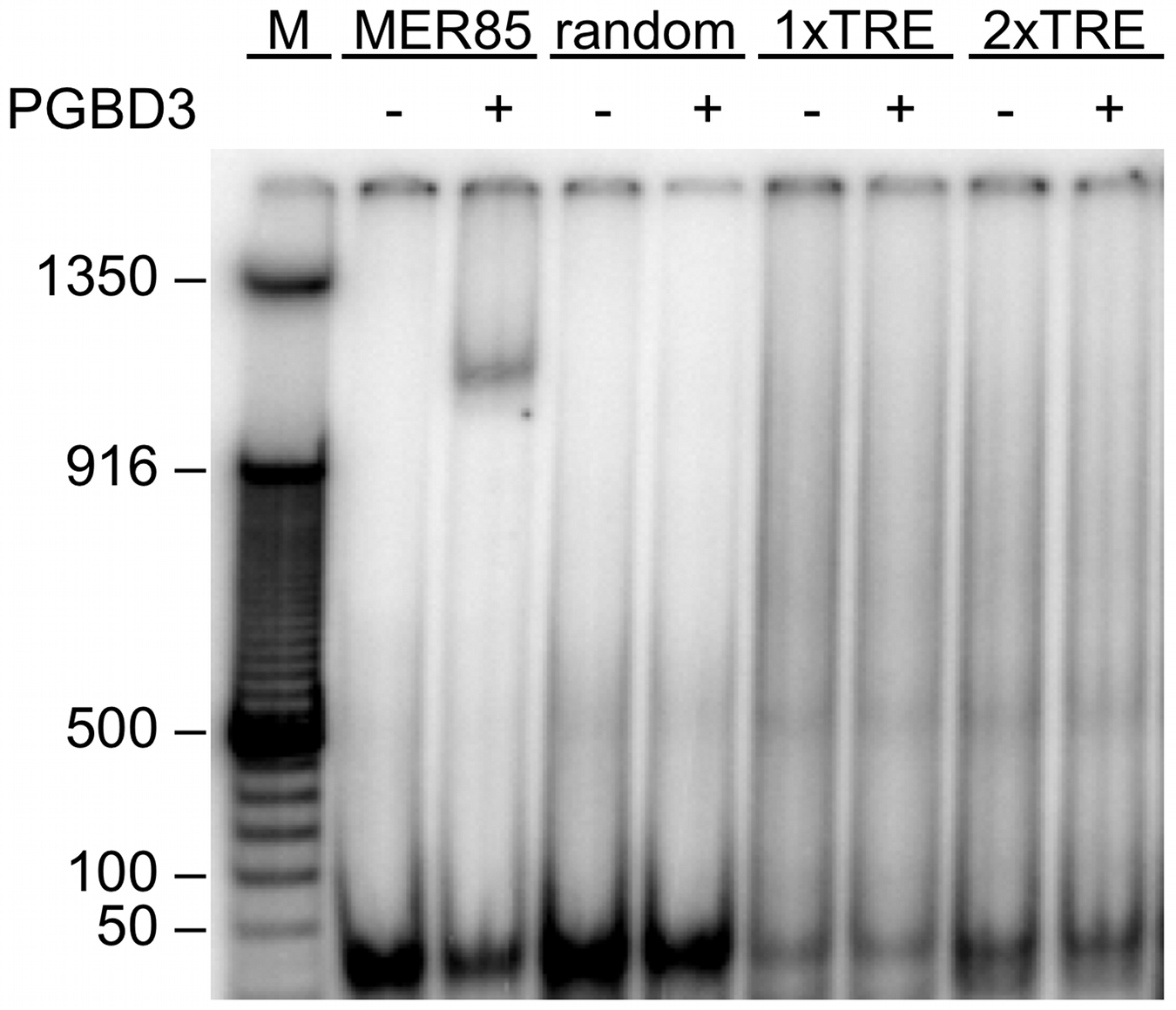

Supplement: Figure S1 — The PGBD3 transposase is not capable of binding directly to TRE motifs in vitro. For EMSA assays, purified PGBD3 transposase was mixed with end-labeled 42 bp duplex oligonucleotides containing Repbase consensus MER85 sequence, 1 or 2 tumor promoting antigen response element (TRE) motifs, or random sequence. (TIF) [file pgen.1002972.s001.tif]

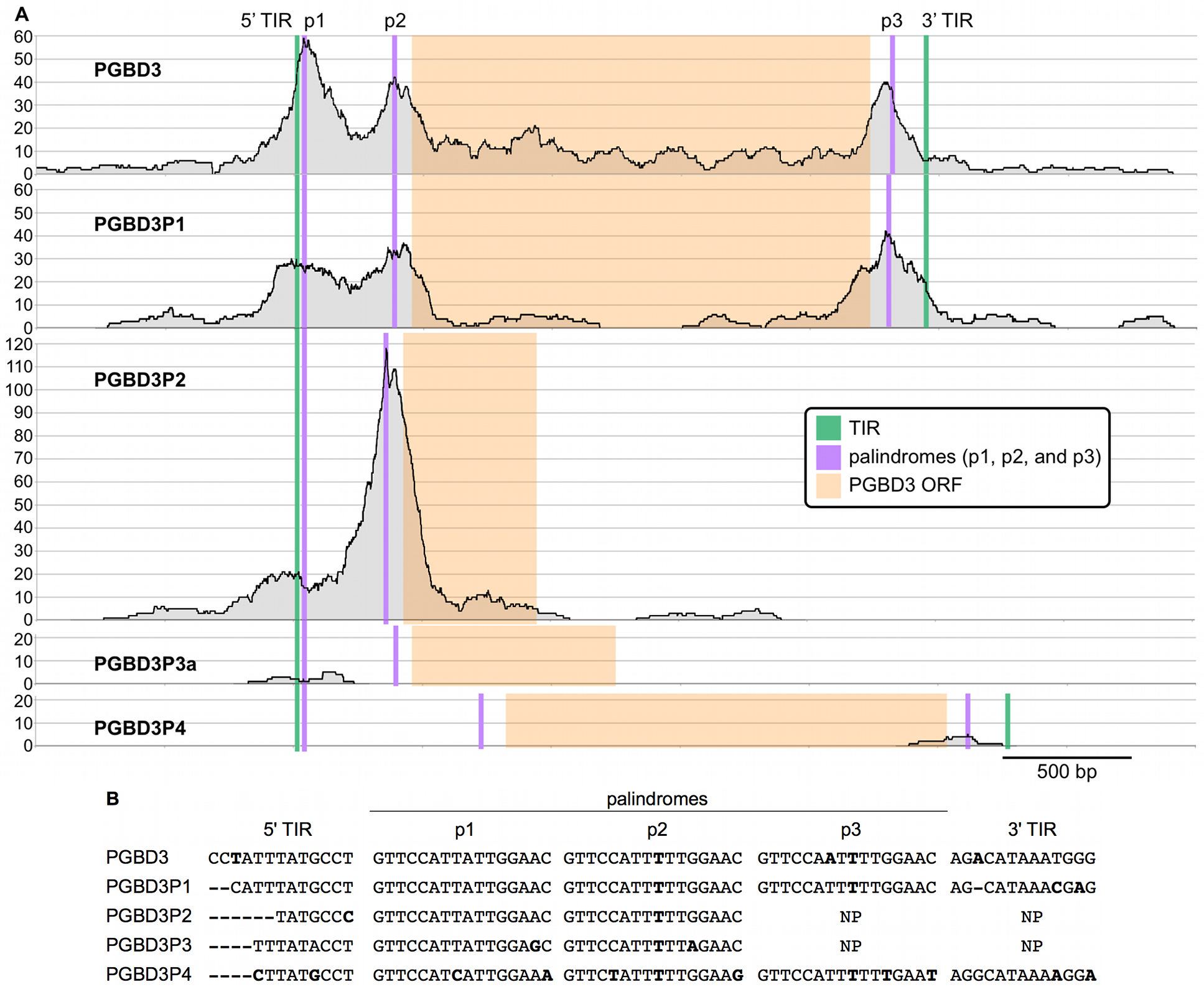

Supplement: Figure S2 — Fragment overlaps over all full-length PGBD3 insertions in the genome, including all four PGBD3 pseudogenes, correlate with conserved palindrome sequences. (a) Fragment overlap binding profiles over PGBD3 and each of the PGBD3 pseudogenes. (b) Sequences of the TIR and palindromes of each of PGBD3 and each of the pseudogenes. Mismatches with respect to the PGBD3 p1 sequence are in bold. TIR, terminal inverted repeat; NP, sequence not present in truncated PGBD3 pseudogene. (TIF) [file pgen.1002972.s002.tif]

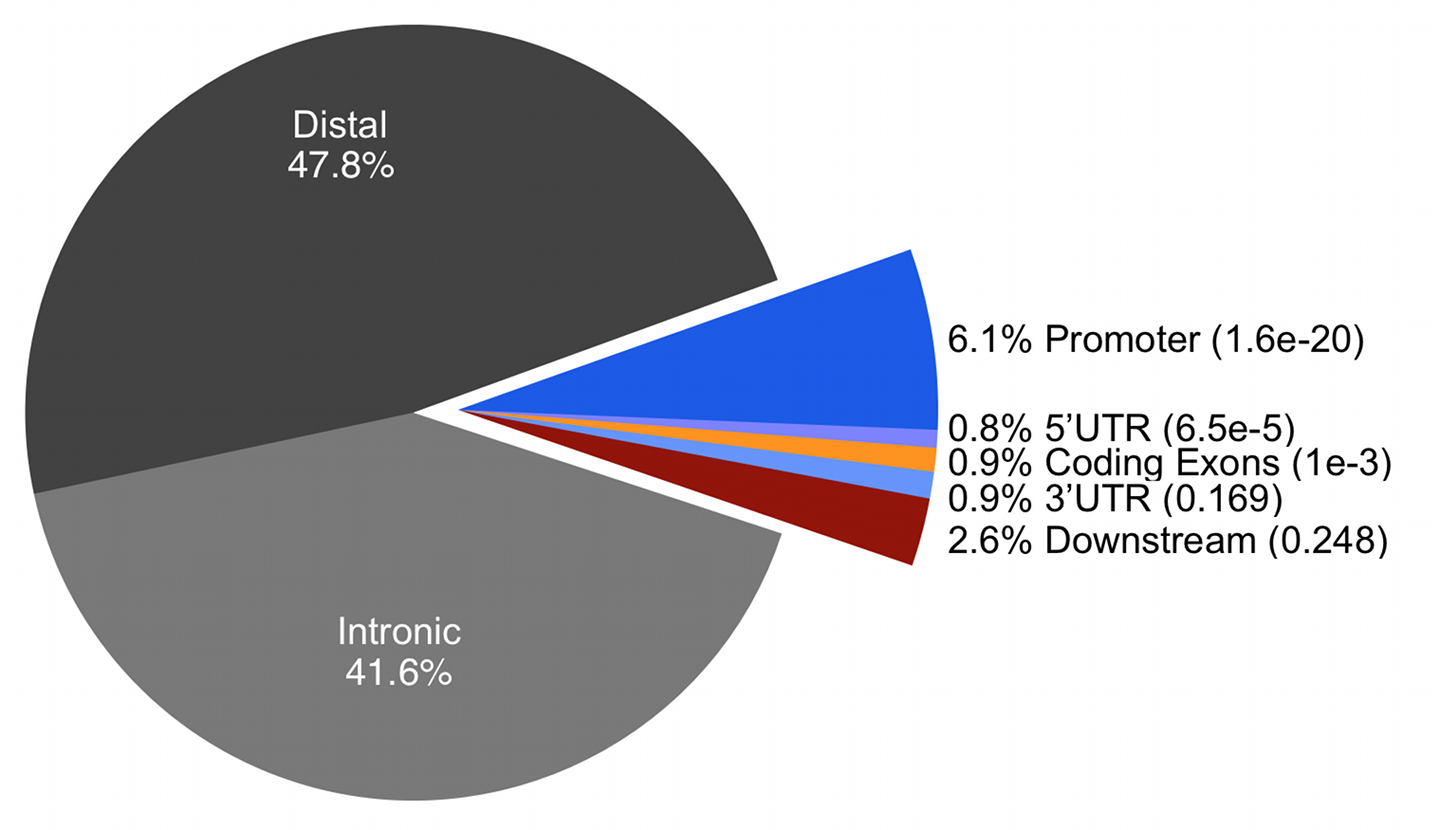

Supplement: Figure S3 — CSB-PGBD3 fusion protein is enriched near gene promoters, but most peaks are distal and intronic. The Cis-regulatory Element Annotation (CEAS) Tool was used to generate a gene-centered annotation of 2,087 CSB-PGBD3 peaks found in common by MACS, ERANGE, and QuEST. Promoter regions include 3 kb upstream of the transcription start site (TSS). Downstream regions include 3 kb beyond the polyadenylation site. P-values generated by CEAS for overrepresentation of CSB-PGBD3 binding are shown in parentheses. (TIF) [file pgen.1002972.s003.tif]

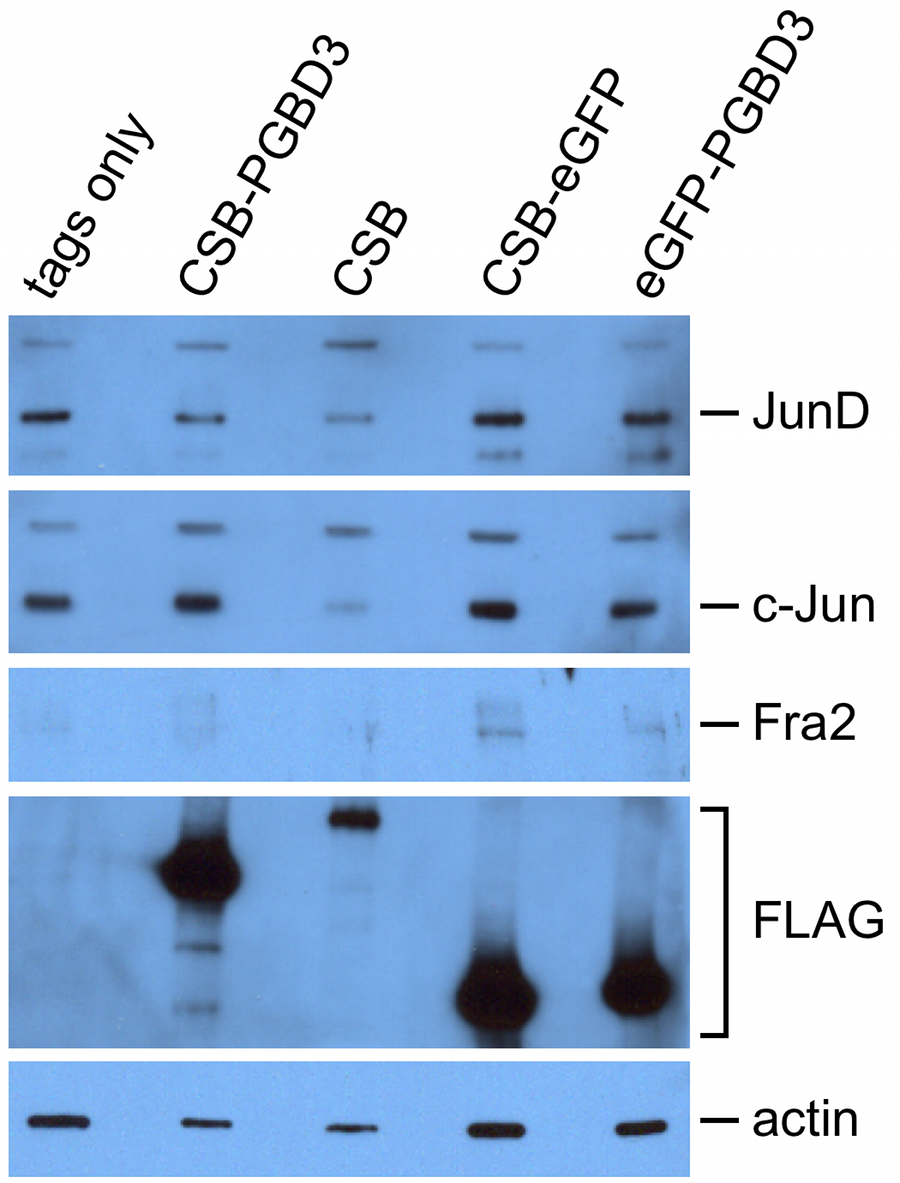

Supplement: Figure S4 — c-Jun, JunD, and Fra2 are expressed in UVSS1KO cell lines. Lysates from UVSS1KO cells expressing FLAG-HA tags or FLAG-HA-tagged CSB-PGBD3, CSB, CSB-eGFP, or eGFP-PGBD3 were western blotted for expression of JunD, c-Jun, Fra2, actin and FLAG-HA-tagged proteins. (TIF) [file pgen.1002972.s004.tif]

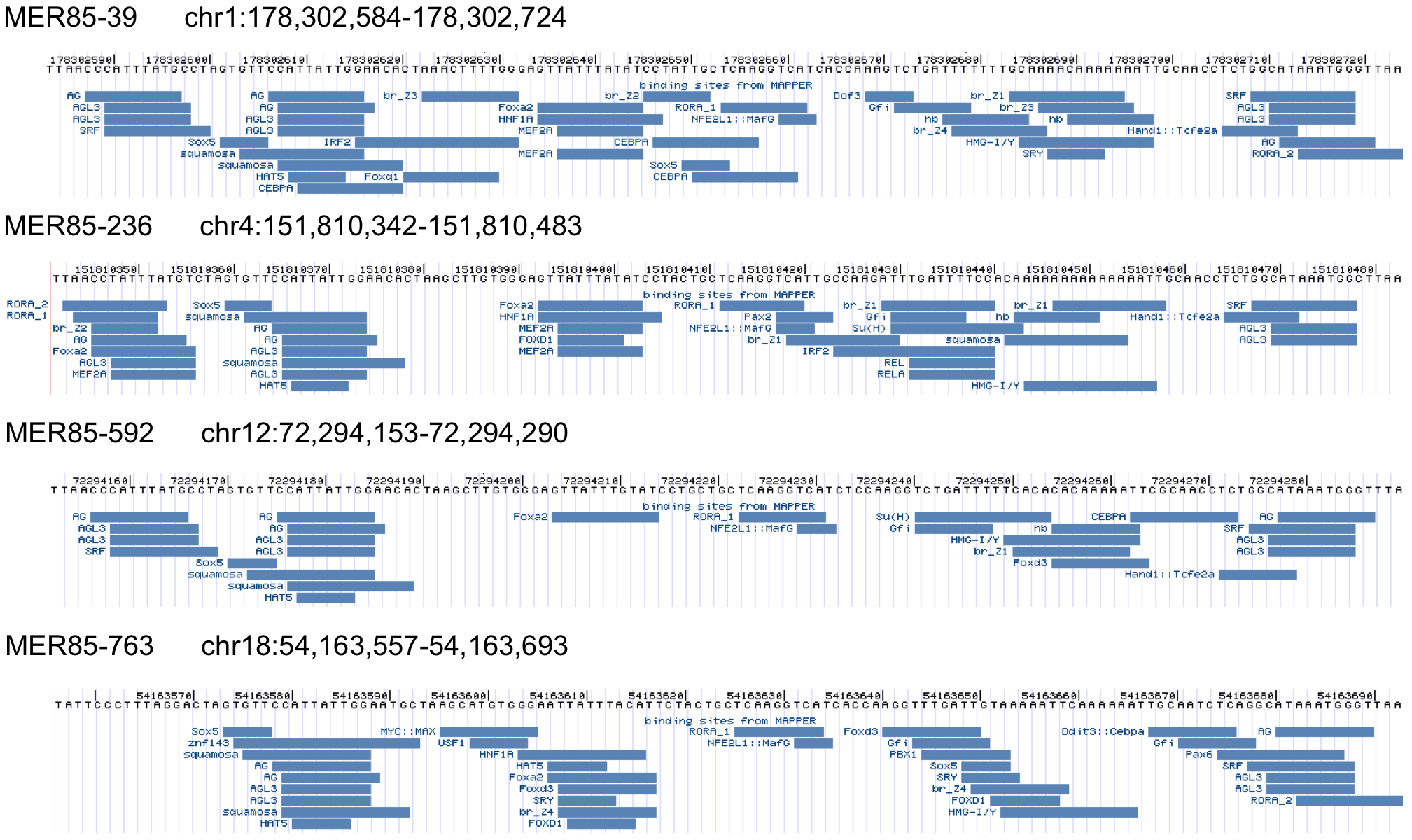

Supplement: Figure S5 — MER85 elements contain potential transcription factor binding sites. Locations of transcription factor binding sites from the JASPAR database found in 4 MER85 elements using MAPPER2 and displayed in the UCSC Genome Browser. MER85 sequences are shown from the upstream to downstream target site duplication (TSD). (TIF) [file pgen.1002972.s005.tif]
